# Generation of a Ground‐State Electron Donor Utilizing Stored Light Energy

**DOI:** 10.1002/chem.70795

**Published:** 2026-02-17

**Authors:** Marc Taillefer, Éric Clot, Alexis Prieto

**Affiliations:** ^1^ ICGM Univ Montpellier, CNRS, ENSCM Montpellier France

**Keywords:** diols, electron donor, light energy storage, reduction, radical

## Abstract

This study presents a process for generating potent ground‐state electron donors using chemically stored light energy. Our strategy leverages light energy through high‐energy vicinal diols produced by the photodimerization of diaryl ketones. Under basic conditions, these diols can produce dianions with potentials near −2.7 V, which further act as strong organic reductants. These reductants demonstrate a high capacity for effectively reducing recalcitrant substrates at room temperature, including electron‐rich aryl bromides, alkyl iodides, and sulfonamide compounds. Mechanistic investigations, supported by density functional theory (DFT) calculations, confirm both the formation of these dianions and their exceptional electron‐donating properties.

## Graphical abstract

**Chemically stored light energy** enables the generation of **dianion reductants** (∼−2.7 V) from vicinal diols derived from diaryl ketone photodimerization. These ground‐state electron donors efficiently reduce challenging substrates at room temperature, including aryl bromides, alkyl iodides, and sulfonamides.

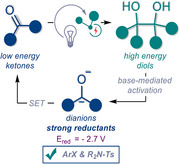

## 1 Introduction

**FIGURE 1:**
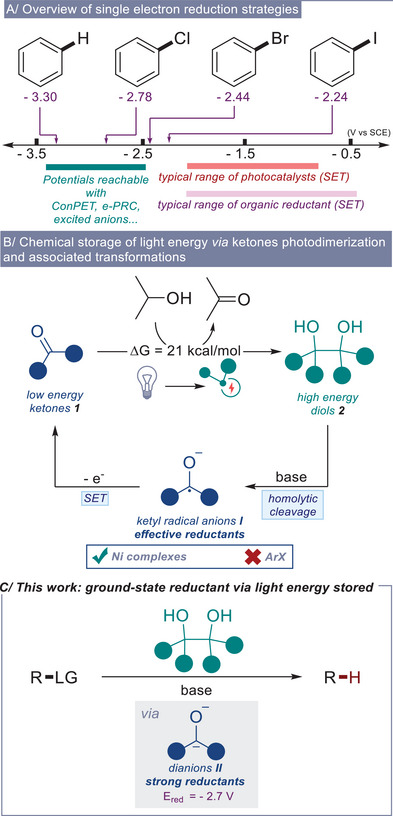
(A) Redox windows for the one‐electron reduction of aryl derivatives. (B) Photodimerization of diaryl ketones and their applications in reductive processes. (C) The generation of ground‐state electron donors via chemically stored light energy.

The redox activation of organic molecules through single‐electron transfer (SET), particularly reductive activation, is a fundamental step in a plethora of useful organic transformations [1, 2, 3, 4]. Historically, such transformations were confined to regimes involving alkali metals, transition metal complexes, or electrochemical methods (Figure 1A) [5]. In recent years, the field of SET reduction has seen significant advancements, with innovative strategies emerging for the activation of substrates under milder reaction conditions. Initially, these transformations were based on the use of ground‐state organic reductants, whether formed *ex‐situ* or *in‐situ* [2, 6, 7, 8, 9, 10, 11, 12, 13, 14, 15], or photocatalysts [16]. However, the use of ground‐state electron donors generally requires elevated temperatures, and both of these methods are mainly narrowed to the activation of compounds with reduction potentials greater than –2.0 V, limiting their applicability to “activated” substrates. More recently, novel procedures that harness light energy have emerged, enabling now the activation of recalcitrant or inert organic molecules. These approaches mainly involve mechanisms such as the photoexcitation of anionic compounds [17, 18, 19, 20, 21, 22, 23, 24, 25, 26], the concept of consecutive photoinduced electron transfer (ConPET) [27, 28], electrochemically mediated photoredox catalysis (e‐PRC) [29, 30, 31], or proton‐coupled electron transfer (PCET) [32, 33]. Each of these strategies facilitates the generation of potent excited or ground‐state reductants with high reduction potentials, often lower than −3.0 V (Figure 1A). This capability underscores the remarkable power of light‐driven processes in organic synthesis.

On the other hand, another approach to harnessing light energy involves its chemical storage within organic molecules akin to the biosynthesis process. This is commonly achieved through syntheses that are thermodynamically unfavorable and require energy input to proceed. For instance, the photoisomerization of alkenes (E→Z) [34] and [2+2]‐cycloadditions [35] are representative processes of chemically stored light energy. However, to effectively utilize this stored light energy, these reactions need to be reversible and to form usable high‐energy intermediates, which is not the case for the previously mentioned processes. A particularly interesting process is the photodimerization of diarylketones, forming highly sterically congested, high‐energy diols that stored around 20 kcal/mol of light energy (Figure 1B) [36]. Several synthetic procedures have demonstrated that, under basic conditions, these diols undergo homolytic cleavage. This cleavage converts their stored light energy into electric potential by forming the corresponding ketyl radical anions (**I**) [37, 38, 39]. In the literature, these ketyl radical anions have further been used as single‐electron reductants for the reduction of nickel complexes (Figure 1B) [37, 38]. Although ketyl radical anions derived from diarylketones are effective reductants (−1.2 V < *E*_red_ < −1.7 V) [40, 41], they are far from being capable of the reduction of recalcitrant substrates such as electron‐rich aryl bromides (*E*_red_ = –2.9 V). In this context, we became interested in the generation of the dianion of diarylketones **II** (Figure 1C). Indeed, these species, reported in the literature as indicators for distillation purification [42, 43, 44], are estimated to have potentials near –2.7 V according to our calculations [45]. Based on our estimations, we envisaged that these dianions (**II**) could be *in‐situ* generated from the corresponding diols under basic conditions either by heterolytic cleavage of the strained C─C bonds, or by disproportionation of the generated ketyl radical anions (Figure 1C). Herein, we describe the first synthetic application of diarylketone dianions **II**, which have been used as potent and efficient reductants for the reduction of aryl bromides, activated aryl chlorides, and sulfonamide compounds.

## 2 Results and Discussion

Preliminary studies have focused on the debromination of 4‐bromoanisole **3a**, which has a high reduction potential (*E*_red_ = –2.9 V vs. saturated calomel electrode (SCE)) [28]. After optimizing the reaction, the best reaction conditions were found using one equivalent of diol **2a**, derived from xanthone **1a**, two equivalents of potassium tert‐butoxide in DMSO at room temperature (Table 1, entry 1). Under these conditions, the reduced anisole **4a** was obtained in an excellent nuclear magnetic resonance (NMR) yield of 91%, and xanthone **1a** was recovered in 80% isolated yield. Using only 0.5 equivalent of dimer **2a**, compound **4a** was formed in a moderate yield of 50%, and 45% of the starting reagent **3a** was recovered (Table 1, entry 2). This result is intriguing as it highlights the necessity of having two units of “monomer” for reducing one unit of **3a**. Decreasing the amount of *^t^* BuOK to one equivalent provided the product **4a** in good yield, albeit 20% of **3a** remained unreacted (Table 1, entry 3). A wide variety of bases were tested for the reaction (Table 1, entries 4–5 and Supplementary Information). While similar results were obtained employing *^t^* BuONa or NaH, reaction outcomes with weak inorganic or strong organic bases were completely ineffective. With other solvents, the reaction was much less efficient, yielding in most of the cases the formation of **4a** in poor yields, with the exception of N,N‐diméthylformamide (DMF) (Table 1, entries 6–8). Despite our expectations, the attempt to reduce 4‐chloroanisole failed (*E*_red_ < –2.9 V), forming only trace amounts of **4a** (Table 1, entry 9). The transformation was then explored using other diols **2**, for which the expected reduction potential of dianions **II** should be higher. (**2b**, ca.–2.8 V; **2c**, ca.–3.6 V) [45]. Unfortunately, in the presence of these diols, the reaction is either barely effective or completely ineffective (Table 1, entries 10 and 11). Finally, control experiments showed that both the base and diol **2a** were essential for the reactivity, and that light did not interfere with the process (Table 1, entries 12–14) [46].

**TABLE 1:** Optimization of the debromination reaction.^a^

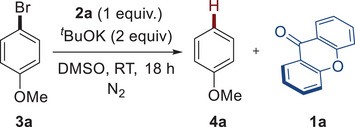			
Entry	Deviations from standard conditions	Yield 4a (%)^b^	Yield 1a (%)
1	None	91	80^c^
2	**2a** (0.5 equiv)	50 (45)^d^	N.D.
3	*^t^* BuOK (1 equiv)	74 (20)^d^	N.D.
4	NaH	82	N.D.
5	CaH_2,_ DBU, or K_3_PO_4_	N.R.	N.D.
6	ACN	25	N.D.
7	DMF	60	N.D.
8	Tetrahydrofuran (THF)	30	N.D.
9	**3a**‐Cl	Trace	N.D.
10	**2b**	14 (84)^d^	N.D.
11	**2c**	N.R.	N.D.
12	Without **2a**	N.R.	N.D.
13	Without *^t^* BuOK	N.R.	N.D.
14	Dark	89	N.D.
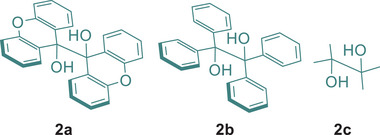			

Abbreviations: N.R., no reaction; N. D., not determined.

^a^ Reaction conditions: **3a** (0.3 mmol), **2a** (0.3 mmol), *^t^* BuOK (0.6 mmol) in DMSO (3 mL).

^b^ Yields were determined by ^1^H NMR using 1,3,5‐trimethoxybenzene as internal standard.

^c^ Isolated yield.

^d^ Remaining starting material **3a**.

**SCHEME 1:**
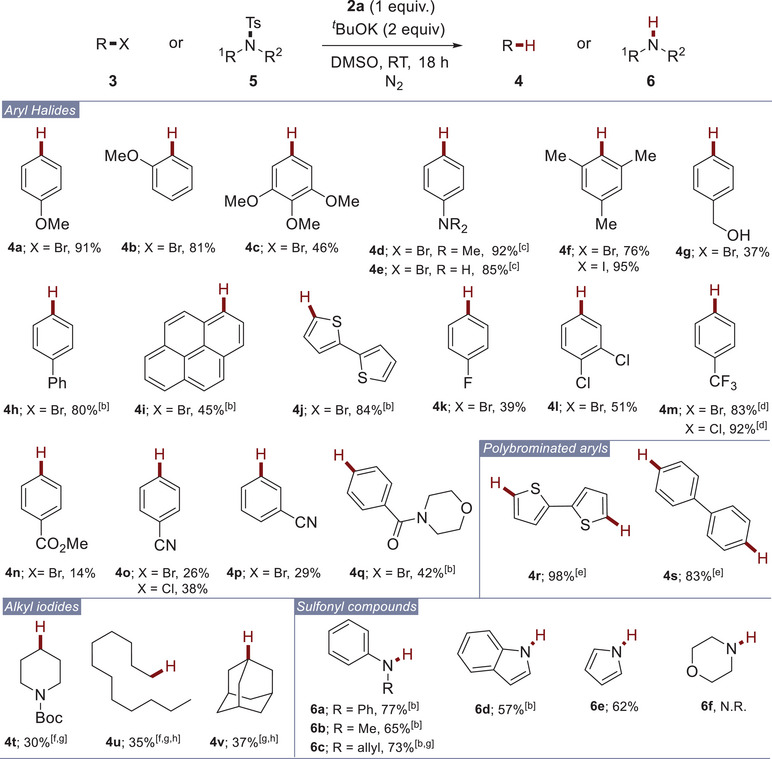
Scope of the dehalogenation of organic halides and desulfonylation of sulfonamides^a^. [a] Reaction conditions: **3** (0.3 mmol), **2a** (0.3 mmol), *^t^* BuOK (0.6 mmol) in DMSO (3 mL). Yields were determined by ^1^H NMR using 1,3,5‐trimethoxybenzene or trichloroethylene as an internal standard in two experiments. [b] Yields refer to isolated products. [c] **2a** (1.25 equiv), *^t^* BuOK (2.5 equiv). [d] Yield was determined by ^19^F NMR using perfluorobenzene or a, a, a‐trifluorotoluene as internal standard. [e] **2a** (2 equiv), *^t^* BuOK (4 equiv). [f] Compound was formed along with its alkene counterpart. [g] NaH (2 equiv) was used as base. [h] Yield was determined by 19F NMR using perfluorobenzene or a,a,a‐trifluorotoluene as internal standard.

With the optimized conditions established, we then explored the scope of the reaction using various electrophiles such as aryl and alkyl bromides, aryl chlorides, alkyl iodides, and *N*‐sulfonylated compounds, which are difficult to reduce via one‐electron reduction (Scheme 1). We initially focused on the reduction of electron‐rich aryl bromides. Pleasingly, our system demonstrated to be productive for reducing various substrates bearing methoxy, amino, and methyl groups, regardless of their positions on the aromatic ring. This led mainly to the formation of reduced compounds **4a–f** in moderate to excellent yields. Interestingly, the observed reactivity with aryl bromide **3f** allows us to rule out any involvement of aryne intermediates in the reaction mechanism, which could form under basic conditions [47]. Additionally, electron‐rich aryl bromide bearing a native alcohol (**3g**) was also suitable for the reduction, yielding the reduced product **4g** in moderate yield (Scheme 1). Electron‐neutral bromo arenes and heteroarenes were also well reduced under our conditions, delivering products **4h–j** in moderate to good yields. However, the reactivity using electron‐poor bromoarenes was mitigated. Indeed, while the reaction furnished products functionalized with halides or a trifluoromethyl group in moderate to excellent yields (**4k–m**), the products bearing ester and cyano functionalities were obtained in poor yields (**4n–p**). This outcome may result from the formation of dimsyl potassium under the established conditions, which is known to condense with electrophiles [48, 49]. However, amide groups were well tolerated, offering the product **4q** in moderate yield. The reactions also demonstrated suitability for the bis‐reduction of polybrominated arenes (**3r–s**) by doubling the amount of diol **2a** and tBuOK (Scheme 1). Intriguingly, while the dehalogenation of electron‐rich chloroarenes such as 4‐chloroanisole or 4‐chlorotoluene failed, the reaction was effective for reducing electron‐poor substrates, yielding the corresponding products **4m** and **4o**, respectively, in excellent and moderate yields. Unfortunately, the reaction proved ineffective for the reduction of alkyl bromides. Indeed, under established reaction conditions, the formation of the corresponding alkenes was ineluctable, likely due to an elimination process. This behavior was typically observed with substrates such as (2‐bromoethyl)benzene or 6‐phenoxyhexyl bromide. Pleasingly, the reaction was moderately effective for reducing alkyl iodides, providing compounds **4t–v** in moderate yields (Scheme 1). However, for substrates **3t** and **3u**, which are prone to elimination, the reaction also formed the corresponding alkenes, with yields below 30% and 10%, respectively. The effectiveness of our method in dehalogenation reactions pushed us to investigate its efficiency for the deprotection of various sulfonyl protecting groups. Indeed, conventional methods for desulfonylation typically require harsh conditions [50]. Moreover, to date, only a few existing ground‐state electron donors can promote this transformation at room temperature [6]. Therefore, our system was studied on a small set of sulfonamides **5** (Scheme 1). Pleasingly, the reaction was effective with several *N*‐tosylanilines, bearing *N*‐phenyl, *N*‐methyl, or *N*‐allyl groups, yielding the corresponding substrates in good yields (**6a–c**). The reaction also worked well on sulfonated *N‐*heterocycles such as indole (**5d**) or pyrrole (**5e**). However, the latter remained non productive for the desulfonylation of *N*,*N*‐dialkylamines, such as morpholine (**5f**).

**SCHEME 2:**
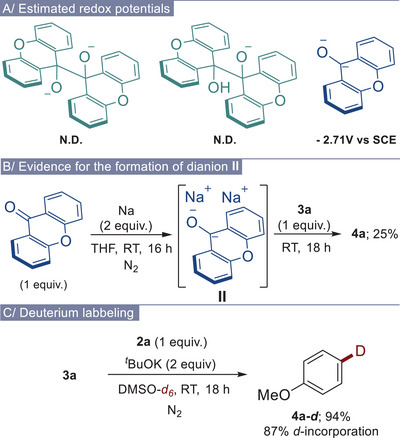
Mechanistic investigations.

Next, we focused on the mechanistic investigation to understand the species potentially involved in this process. First, data obtained during the optimization part, especially the reaction using only 0.5 equivalent of diol **2a** (Table 1, entry 2), clearly indicated that two units of xanthone are mandatory for reducing compound **3a**. This suggests that the electron donation likely occurs either from a dimeric form (bis‐ or mono‐deprotonated diol **2a**, Scheme 2A) or from a monomeric xanthone derivative generated from two units of xanthone (dianion **II**, Scheme 2A). Therefore, to determine which species is the electron donor, we decided to determine their redox potentials by density functional theory (DFT) calculations. While we successfully estimated the potential of the dianion **II** at –2.71 V (Scheme 2A) [45], we were unable to obtain the potentials of the dimeric species. Their oxidized forms could not be optimized under DFT calculations due to homolytic cleavage of the dimer (vide infra). Although these findings do not rule out the involvement of dimeric species, they strongly suggest that the xanthone dianion **II** can be the ground‐state donor responsible for reducing compounds **3** and **5**. To get further evidence for the formation and the role of dianion **II**, we then examined the reaction using xanthone and sodium metal (Scheme 2B). Remarkably, when the reduction of aryl bromide **3a** was investigated using two equivalents of sodium in association with one equivalent of xanthone in THF, the reduced compound **4a** was obtained in 25% (Scheme 2B) [51]. This result is comparable to those previously obtained in this solvent (Table 1, entry 8). Interestingly, when the reaction was conducted using only one equivalent of sodium, the starting bromide **3a** was fully recovered, and no formation of **4a** was detected. These results highly support the formation of dianion **II** and its role as the active electron donor in the reaction. To gain further insights into the operating mechanism, deuterium labeling and radical trapping experiments were also conducted (Scheme 2 and see supplementary information). When the debromination of **3a** was carried out in DMSO‐*d_6_*, the product **4a** was obtained in nearly quantitative yield with 87% of deuterium incorporation (Scheme 2C). This supports the idea that DMSO acts as a hydrogen donor in the final hydrogen atom transfer step.

**SCHEME 3:**
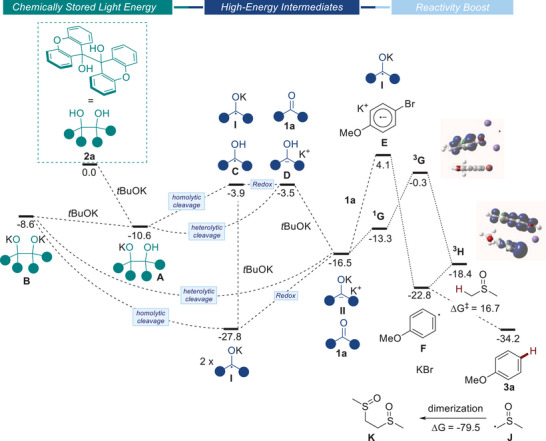
DFT calculations computed at the PBE0‐def2‐qzvp, SMD(DMSO) level of theory. Values are given in kcal/mol.

For the DFT calculations, diol **2a** and 4‐bromoanisole (**1a**) were chosen as model substrates (Scheme 3). The geometries were optimized at the PBE0‐D3(bj) level with implicit inclusion of the solvent (SMD model DMSO) in the optimization process (see Supplementary Information for more details). Initially, we examined the deprotonation of diol **2a** in the presence of *^t^* BuOK. The first deprotonation step is exergonic (Δ*G* = –10.6 kcal/mol^−1^), yielding intermediate **A**. However, the subsequent deprotonation of **A**, leading to intermediate **B**, is slightly endergonic (Δ*G* = 2.0 kcal/mol^−1^). From intermediates **A** and **B,** the homolytic and heterolytic cleavage of the C─C bond might both be considered. *Intermediate* ***A***: While the heterolytic cleavage would yield intermediate **D** and ketone **1a** (Δ*G* = 7.1 kcal/mol^−1^), the homolytic cleavage, which is endoergic (Δ*G* = 6.7 kcal/mol^−1^), would lead to the formation of the ketyl radical anion **I** and the intermediate **C**. At this stage, two new pathways are possible: (a) deprotonation of **C**, furnishing another equivalent of ketyl radical anion **I** (Δ*G* = −23.9 kcal/mol^−1^), (b) or the redox reaction between **I** and **C**, affording intermediate **D** and ketone **1a** (Δ*G* = −0.4 kcal/mol^−1^). The deprotonation of **D** is computed to be highly exoergic (Δ*G* = −13 kcal/mol^−1^), forming the corresponding dianion **II**. Interestingly, the comproportionation process of ketone **1a** and dianion **II**, delivering two units of ketyl radical anion **I,** is exoergic (Δ*G* = −12.3 kcal/mol^−1^, redox, Scheme 3). *Intermediate* ***B***: The latter might be involved in both of the above‐mentioned mechanistic scenarios. Exoergic homolytic cleavage (Δ*G* = −19.2 kcal/mol^−1^) would deliver two units of ketyl radical anion **I**. Similarly, heterolytic cleavage, also exoergic (Δ*G* = −6.9 kcal/mol^−1^), would generate ketone **1a** and dianion **II** that can further undergo comproportionation to form two ketyl radical anions **I**. All the DFT calculations support the formation of ketyl radical anions **I** that remain stable and accumulate in solution (as observed experimentally). We propose that this stability is crucial for achieving the endoergic disproportionation (2 × **I**→**II **+ **1a**; Δ*G* = 11.3 kcal/mol^−1^). Following this, the formation of the aryl radical **F** is computed to be exoergonic (Δ*G* = −6.3 kcal/mol^−1^). This transformation could potentially occur sequentially with first endoergic reduction of the aryl bromide to form the radical anion **E** (Δ*G* = 20.6 kcal/mol^−1^), followed by exoergic dissociation of Br^−^ (Δ*G* = −26.9 kcal/mol^−1^) to form **F**. Alternatively, the formation of the aryl radical **F** could occur in a concerted reduction of the aryl bromide/bromide dissociation. To probe the energetics associated with such a pathway, the formation of the adduct (singlet state) **^1^G** between **II** and the aryl bromide is computed to be endoergic by only Δ*G* = 3.2 kcal/mol^−1^. The corresponding adduct in the triplet state, **^3^G**, lies at Δ*G* = 16.2 kcal/mol^−1^, and the spin density of this state is fully developed on the **II** fragment (Scheme 3). Dissociation of KBr from **^3^G** to form the adduct **^3^H** between the aryl radical **F** and the ketyl radical **I** is computed to be exoergic (Δ*G* = −18.1 kcal/mol^−1^). The spin density of **^3^H** clearly features a radical nature at the aromatic ring (Scheme 3). Despite numerous attempts, no transition state for the concerted intra‐adduct electron transfer and bromide dissociation from **^3^G** to **^3^H** could be located on the potential energy surface. However, the calculations are more in favor of a concerted process associated with lower lying intermediates. Finally, dissociation of the aryl radical **F** in **^3^H** opens a pathway for hydrogen atom transfer (Δ*G* ^‡^ = 16.7 kcal/mol^−1^, Δ*G* = −11.4 kcal/mol^−1^) from DMSO to form **3a** alongside radical **J**. The radical **J** would finally dimerize to provide **K**. Overall, DFT calculations give robust support for the light storage concept: the photodimerization of **1a** affords high‐energy diol **2a**, which can further deliver high‐energy intermediates (species **I** and **II**). These intermediates enhance reactivity before returning to ketone **1a** (Scheme 3).

## 3 Conclusion

In conclusion, we report the first strategy that harnesses light energy by chemically storing it, which can further be employed to generate a potent ground‐state electron donor. The latter is capable of reducing various electron‐rich aryl bromides, electron‐poor chlorides, and *N*‐sulfonyl compounds at room temperature. Based on DFT calculations, the donor is assumed to be the dianion of xanthone, exhibiting an exceptional reduction potential of –2.7 V that outperforms most other reported ground‐state donors. Additionally, these calculations afford a comprehensive mechanistic overview for the formation of xanthone dianion. This study constitutes the first example of the synthetic utility of diaryl ketone dianions, which were previously only used as distillation indicators. Our findings open new opportunities for the development of transformations employing such unconventional reductants.

## Conflicts of Interest

The authors declare no conflicts of interest.

## Supporting information




**Supporting File 1**: Additional supporting information can be found online in the Supporting Information section. The supporting information (SI) contains the experimental procedures, optimization table (Table S1), list of unsuccessful substrates, characterization of compounds **4** and **6**, Picture of reaction evolution (Figure S1), Redox potential evaluation, mechanistic investigations, computational details, and NMR spectra. Additional references cited within the Supporting Information [1–20].
