# Supplementary material for: Generation of a Ground‐State Electron Donor Utilizing Stored Light Energy
Source: Chemistry. 2026 Feb 17;32(17):e70795. doi: 10.1002/chem.70795 (PMC13150042; doi:10.1002/chem.70795)
Supplement: Supplementary file 1 — Supporting File 1: Additional supporting information can be found online in the Supporting Information section. The supporting information (SI) contains the experimental procedures, optimization table (Table S1), list of unsuccessful substrates, characterization of compounds 4 and 6, Picture of reaction evolution (Figure S1), Redox potential evaluation, mechanistic investigations, computational details, and NMR spectra. Additional references cited within the Supporting Information [1–20]. [file CHEM-32-e70795-s001.docx]

**Supporting Information**

**Generation of a ground-state electron donor utilizing stored light energy**

Marc Taillefer, Éric Clot, Alexis Prieto*

ICGM, Univ Montpellier, CNRS, ENSCM, 34296 Montpellier, France

E-mail: alexis.prieto@enscm.fr

**Table of content**

[A. General information 2](#_Toc220569170)

[B. Synthesis of compound **2a**^1^ 2](#_Toc220569171)

[C. Optimization for reduction of aryl bromides 3](#_Toc220569172)

[D. List of unsuccessful compounds 4](#_Toc220569173)

[E. Characterization of compounds **4** and **6** 4](#_Toc220569174)

[F. Redox potential evaluation 6](#_Toc220569175)

[G. Mechanistic investigations 7](#_Toc220569176)

[H. Computational Details 10](#_Toc220569177)

[I. References 10](#_Toc220569178)

[J. Crude NMR Spectra of volatile compounds **4** and **6** 12](#_Toc220569179)

[K. NMR Spectra of compounds **4** and **6** 18](#_Toc220569180)

# **General information**

^1^H, ^13^C and ^19^F spectra were recorded on a Bruker®AC-400 MHz spectrometer (400 MHz, 101 MHz, and 376 MHz respectively) at ambient temperature. The peaks were internally referenced to residual undeuterated solvent signal (CDCl_3_: 7.26 ppm (^1^H NMR), 77.16 ppm (^13^C NMR)). The following abbreviations were used to explain multiplicities: s = singlet, d = doublet, t = triplet, q = quadruplet, m = multiplet, br = broad). All ^19^F NMR spectra are reported in ppm relative to CFCl_3_. NMR yields were determined by using respectively trifluorotoluene or trifluoroanisole for ^19^F NMR yields and trichloroethylene for ^1^H NMR yields as internal standards. HRMS (Q-TOF) studies were performed on a JEOL JMS-DX300 spectrometer (3 keV, xenon) in a m-nitrobenzylalcohol matrix. All reactions were performed under an argon atmosphere in a sealed reaction vial. Reactions were monitored by analytical thin layer chromatography (TLC) using commercial sheets precoated (0.2 mm layer thickness) with silica gel 60F254 (Macherey-Nagel). Product purification by flash column chromatography was performed using Macherey-Nagel Silica Gel (40-63 μm). Solvents and reagents were obtained from commercial sources and used as received.

**Materials.** Commercial grade reagents and solvents were purchased from Sigma-Aldrich, Fluka, Alfa Aesar, Fluorochem, SynQuest at the highest commercial quality and used without further purification, unless otherwise stated. Starting materials **2a**,^[[1]](#endnote-1)^ **2b**^[[2]](#endnote-2)^ and **5a-5f**^[[3]](#endnote-3)^ were synthesized according to described procedures.

# Synthesis of compound 2a^1^

To a round bottom flask was added a stir bar, xanthone (10.0 g, 51.0 mmol, 1.00 equiv.) along with 2-propanol (1.02 L, 0.05 M) and 10 drops of glacial acetic acid. The vessel was then sealed with a septum, and the solution was degassed with N_2_ for 30 minutes. The vessel was irradiated with two Kessil UV LED lamps (390 nm) and stirred for 48 hours. Upon completion, the reaction mixture was then concentrated in vacuo. The crude mixture was then filtered via vacuum using diethyl ether (3 × 15 mL), followed by hexane (2 × 15 mL). The solid was then allowed to be dried under high vacuum overnight. The desired compound was obtained as a light yellow solid (7.4 g, 74%). ***Compound 2a is bench-stable and can be stored for several months without any observable degradation.***

# Optimization for reduction of aryl bromides

| Entry | Deviations | Yield of **3a** (%)^[b]^ | Yield of **1a** (%)^[b]^ |
| --- | --- | --- | --- |
| 1 | none | 91 | 80^[c]^ |
| 2 | **2a** (0.5 equiv.) | 50 (45)^[d]^ | / |
| 3 | *^t^*BuOK (1 equiv.) | 74 (20)^[d]^ | / |
| 4 | *^t^*BuONa (2 equiv.) | 88 | / |
| 5 | NaH (2 equiv.) | 82 | / |
| 6 | CaH_2,_ DBU, or K_3_PO_4_ (2 equiv.) | N.R. | / |
| 7 | TBD (2 equiv.) | N.R. | / |
| 8 | BTPP (2 equiv.) | 0 (80)^[d]^ | / |
| 9 | ACN | 25 | / |
| 10 | DMF | 60 | / |
| 11 | EtOAc | 10 | / |
| 12 | THF | 30 | / |
| 13 | **3a**-Cl | trace | / |
| 14 | without **2a** | 0 | / |
| 15 | **2b** | 14 (84)^[d]^ | / |
| 16 | **2c** | N.R. | / |
|   [a] Reaction conditions: **3a** (0.3 mmol), **2a** (0.3 mmol), *^t^*BuOK (0.6 mmol) in DMSO (3 mL). [b] Yields were determined by ^1^H NMR using 1,3,5-trimethoxybenzene as internal standard. [c] Isolated yield. [d] Remaining starting material **3a**. N.R. = No reaction. | | | |

**Table S1.** General optimization of 4-bromoanisole reduction.

# List of unsuccessful compounds

Under the optimal conditions several compounds were found unreactive or led to the formation of other products, see structure below.

# Characterization of compounds 4 and 6

**General Procedure 1 (GP1)**. (*0.3 mmol scale reaction*). In a vial, potassium *tert*-butoxide (67.4 mg, 0.6 mmol, 2 equiv.) was added to a solution of the selected compound **3** or **5** (0.3 mmol, 1 equiv.) and the xanthopinacol **2a** (118.4 mg, 0.3 mmol, 1 equiv.) in DMSO (3 mL). The reaction mixture was degassed v*ia* a freeze pump thaw procedure, and then stirred at room temperature (22-25°C) for 18 h.

***For volatile products***: NMR yields were determined as follows (products **4a**-**4g**, **4k**-**4o**): Crude mixture was quenched with 3 mL H_2_O and 1 mL of brine. Then 1,3,5-trimethoxybenzene (50.4 mg, 0.3 mmol, 1 equiv.) or trichloroethynene (27 µL, 0.3 mmol, 1 equiv.) was added as the internal standard to the crude reaction mixture. Finally, the mixture was extracted with 1-2 mL of CDCl_3_ and the reactions were analyzed *via* ^1^H NMR of the CDCl_3_ layer. The crude mixtures were also analyzed by GCMS analysis.

***For non-volatile products:*** After reaction was completed, the reaction mixture was transferred to an extraction funnel, 10 mL of H_2_O and 2 mL of brine were added and the organic layer was extracted with DCM. The organic layer was washed twice with brine, the combined organic layers were dried over anhydrous MgSO_4_, filtered and concentrated using a rotary evaporator. The crude residue was purified by column chromatography to afford the corresponding products **4** or **6**.

Typically observed colors are shown in the **Figure S1**. An initial ‘red’ color was observed for several substrates (mostly electron-poor substrates), before turning green-blue.


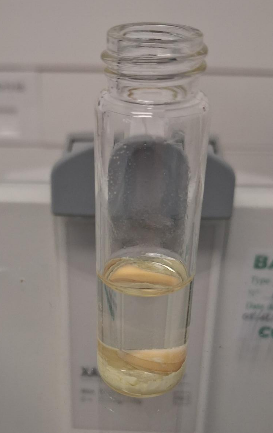

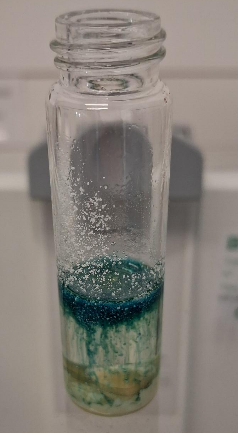

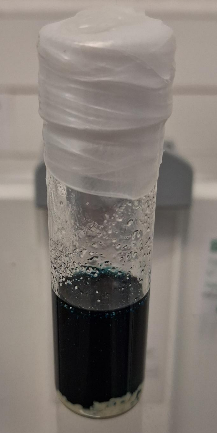


**Figure S1.** Left: Reaction mixture before the addition of t-BuOK. Center: Reaction mixture just after the addition of t-BuOK. Right: Reaction mixture 2 minutes after the addition of t-BuOK.

****1,1’-biphenyl (4h):** Following **GP1** and purified by silica gel column chromatography (100% PE to 95:5 PE/DCM) to give the corresponding product as a white solid (27 mg, 45 %). **^1^H NMR** (400 MHz, CDCl_3_) δ 7.66 – 7.58 (m, 4H), 7.51 – 7.41 (m, 4H), 7.41 – 7.32 (m, 2H). **^13^C NMR** (101 MHz, CDCl_3_) δ 141.4, 129.0, 127.4, 127.3. **GC-MS** (m/z): 154. Spectroscopic data obtained lare consistent with the data reported in the literature.^[[4]](#endnote-4)^

****pyrene (4i):** Following **GP1** and purified by silica gel column chromatography (100% PE to 95:5 PE/DCM) to give the corresponding product as a white solid (27 mg, 45 %). **^1^H NMR** (400 MHz, CDCl_3_) δ 8.20 (d, J = 7.6 Hz, 4H), 8.10 (s, 4H), 8.03 (dd, J = 8.1, 7.2 Hz, 2H). **^13^C NMR** (101 MHz, CDCl_3_) δ 131.3, 127.5, 126.0, 125.1, 124.8. **GC-MS** (m/z): 202. Spectroscopic data obtained are consistent with the data reported in the literature.^[[5]](#endnote-5)^

****2,2’-bithiophene (4j):** Following **GP1** and purified by silica gel column chromatography (100% PE to 95:5 PE/DCM) to give the corresponding product as a white solid (42 mg, 84 %). **^1^H NMR** (400 MHz, CDCl_3_) δ 7.22 (dd, J = 5.1, 1.2 Hz, 2H), 7.19 (dd, J = 3.6, 1.2 Hz, 2H), 7.03 (dd, J = 5.1, 3.6 Hz, 2H). **^13^C NMR** (101 MHz, CDCl_3_) δ 137.5, 127.9, 124.5, 123.9. **GC-MS** (m/z): 166. Spectroscopic data obtained are consistent with the data reported in the literature.^[[6]](#endnote-6)^

****morpholino(phenyl)methanone (4p):** Following **GP1** and purified by silica gel column chromatography (1:1 PE/AcOEt) to give the corresponding product as a slightly yellow solid (24 mg, 42 %). **^1^H NMR** (400 MHz, CDCl_3_) δ 7.44 – 7.40 (m, 5H), 3.73 (brs, 6H), 3.45 (brs, 2H). **^13^C NMR** (101 MHz, CDCl_3_) δ 170.6, 135.4, 130.0, 128.7, 127.2, 67.0, 48.3 (brs), 42.7 (brs). **GC-MS** (m/z): 191. Spectroscopic data obtained are consistent with the data reported in the literature.^[[7]](#endnote-7)^

****diphenylamine (6a):** Following **GP1** and purified by silica gel column chromatography (100% PE to 9:1 PE/AcOEt) to give the corresponding product as a white solid (39 mg, 77 %). **^1^H NMR** (400 MHz, CDCl_3_) δ 7.32 – 7.21 (m, 4H), 7.11 – 7.03 (m, 4H), 6.98 – 6.88 (m, 2H). **^13^C NMR** (101 MHz, CDCl_3_) δ 143.2, 129.5, 121.2, 118.0. **GC-MS** (m/z): 169. Spectroscopic data obtained are consistent with the data reported in the literature.^[[8]](#endnote-8)^

***N*-methylamine (6b):** Following **GP1** and purified by silica gel column chromatography (1:1 PE/DCM) to give the corresponding product as a slightly yellow oil (21 mg, 65 %). **^1^H NMR** (400 MHz, CDCl_3_) δ 7.26 – 7.13 (m, 2H), 6.85 – 6.69 (m, 1H), 6.69 – 6.59 (m, 2H), 3.64 (bs, 1H), 2.85 (s, 3H). **^13^C NMR** (101 MHz, CDCl_3_) δ 149.4, 129.3, 117.4, 30.9. **GC-MS** (m/z): 107. Spectroscopic data obtained are consistent with the data reported in the literature.^[[9]](#endnote-9)^

***N*-allyllamine (6c):** Following **GP1**, except that NaH was used as base instead of *t*-BuOK, and purified by silica gel column chromatography (100% PE to 9:1 PE/EtOAc) to give the corresponding product as a yellow oil (29 mg, 73 %). **^1^H NMR** (400 MHz, CDCl_3_) δ 7.25 – 7.12 (m, 2H), 6.78 – 6.68 (m, 1H), 6.75 – 6.61 (m, 2H), 5.97 (ddt, J = 17.2, 10.3, 5.4 Hz, 1H), 5.29 (dq, J = 17.2, 1.7 Hz, 1H), 5.17 (dq, J = 10.3, 1.5 Hz, 1H), 3.78 (dt, J = 5.4, 1.6 Hz, 2H). **^13^C NMR** (101 MHz, CDCl_3_) δ 148.0, 135.5, 129.4, 117.9, 116.5, 113.3, 46.9. **GC-MS** (m/z): 133. Spectroscopic data obtained are consistent with the data reported in the literature.^[[10]](#endnote-10)^

**Indole (6d):** Following **GP1** and purified by silica gel column chromatography (100% PE to 9:1 PE/EtOAc) to give the corresponding product as a slightly yellow solid (20 mg, 57 %). **^1^H NMR** (400 MHz, CDCl_3_) δ 8.15 (s, 1H), 7.66 (dq, J = 7.8, 0.9 Hz, 1H), 7.45 – 7.38 (m, 1H), 7.24 – 7.17 (m, 1H), 7.20 – 7.08 (m, 1H), 6.57 (tt, J = 2.0, 1.0 Hz, 1H). **^13^C NMR** (101 MHz, CDCl_3_) δ 135.9, 128.0, 124.3, 122.1, 120.8, 119.9, 111.2, 102.7. **GC-MS** (m/z): 117. Spectroscopic data obtained are consistent with the data reported in the literature.^[[11]](#endnote-11)^

# Redox potential evaluation

All the redox potential were determined following a described procedure^[[12]](#endnote-12)^ and using the flowing equation:


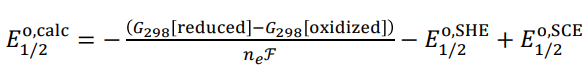


Where n*_e_* is the number of electrons transferred (in all calculations here, n*_e_* = 1 and is accordingly omitted from all subsequent expressions), ℱ is the Faraday constant (value 23.061 kcal mol^-1^V^-1^ ), E_1/2_^0,SHE^ is the absolute value for the standard hydrogen electrode (SHE, value = 4.281 V) and E_1/2_^0,SCE^ is the potential of the saturated calomel electrode (SCE) relative to SHE in acetonitrile (value = - 0.141 V), and G_298_[oxidized] and G_298_[oxidized] are the Gibbs free energies in acetonitrile as gathered from DFT calculations.

**Determination of the redox potential of dianion II-a**

G_298_[oxidized] = - 650.672720 Hartree

G_298_[reduced] = - 650.735732 Hartree

ΔG^0^_1,2_ = (G_298_[reduced] - G_298_[oxidized]) = (- 650.735732 - - 650.672720 Hartree) x 627.5 kcal.mol^-1^.Hartree^-1^ = - 39.54 kcal.mol^-1^

E^0^_1/2_^calc^ = - (-39.54 / 23.061) - 4.281 - 0.141 = **- 2.71 V vs SCE**

**Determination of the redox potential of dianion II-b**

G_298_[oxidized] = - 576.624098 Hartree

G_298_[reduced] = - 576.684111 Hartree

ΔG^0^_1,2_ = (G_298_[reduced] - G_298_[oxidized]) = (- 576.684111 - - 576.624098 Hartree) x 627.5 kcal.mol^-1^.Hartree^-1^ = - 37.66 kcal.mol^-1^

E^0^_1/2_^calc^ = - (- 37.66 / 23.061) - 4.281 - 0.141 = **- 2.79 V vs SCE**

**Determination of the redox potential of dianion II-b**

G_298_[oxidized] = - 193.183824 Hartree

G_298_[reduced] = - 193.213678 Hartree

ΔG^0^_1,2_ = (G_298_[reduced] - G_298_[oxidized]) = (- 193.213678 - - 193.183824 Hartree) x 627.5 kcal.mol^-1^.Hartree^-1^ = - 18.73 kcal.mol^-1^

E^0^_1/2_^calc^ = - (- 18.73 / 23.061) - 4.281 - 0.141 = **- 3.61 V vs SCE**

# Mechanistic investigations

**Experiment with metallic sodium.**

**With 2 equiv. of Na.** In a vial containing a solution of xanthone **1a** (58.8 mg, 0.3 mmol, 1 equiv.) in THF (1 mL) metallic sodium (13.8 mg, 0.6 mmol, 2 equiv.) was added. The reaction mixture was degassed v*ia* a freeze pump thaw procedure, and stirred at room temperature for 16h (overnight). Over this period the solution color gradually changed from pale yellow to blue and finally to purple. Then, substrate **3a** (38 µL, 0.3 mmol, 1 equiv.) was added to the purple solution and stirred for additional 18 h. Under these conditions, reduced compound **4a** was formed in 25% yield.

**Figure S2.** Reaction with 2 equivalents of metallic sodium

**With 1 equiv. of Na.** In a vial containing a solution of xanthone **1a** (58.8 mg, 0.3 mmol, 1 equiv.) in THF (1 mL) metallic sodium (6.9 mg, 0.3 mmol, 1 equiv.) was added. The reaction mixture was degassed v*ia* a freeze pump thaw procedure, and stirred at room temperature for 16h (overnight). Over this period the solution color gradually changed from pale yellow to blue. ***Purple color has never been observed under those conditions***. Then, substrate **3a** (38 µL, 0.3 mmol, 1 equiv.) was added to the solution and stirred for additional 18 h. Under these conditions, the compound **4a** was not detected.

**Figure S3.** Reaction with 1 equivalent of metallic sodium

**Deuterium labeling.** The reaction was set up following the **GP1**, except that deuterated DMSO was used. Under those conditions, compound **4a** was formed in 94% yield with 87% of deuterium incorporation. To obtain these results, 1 equivalent of 1,3,5-trimethoxybenzene was added and used as an internal standard. The integration of the three aromatic protons (H_Ar_) from 1,3,5-trimethoxybenzene was set to 1.00, while the three methoxy protons (H_OMe_) of compound **4a** integrated to 0.94. The doublet integrating to 0.68 corresponds to the 2H of the deuterated compound, and the signal integrating to 0.16 corresponds to the 3H of the non-deuterated compound.

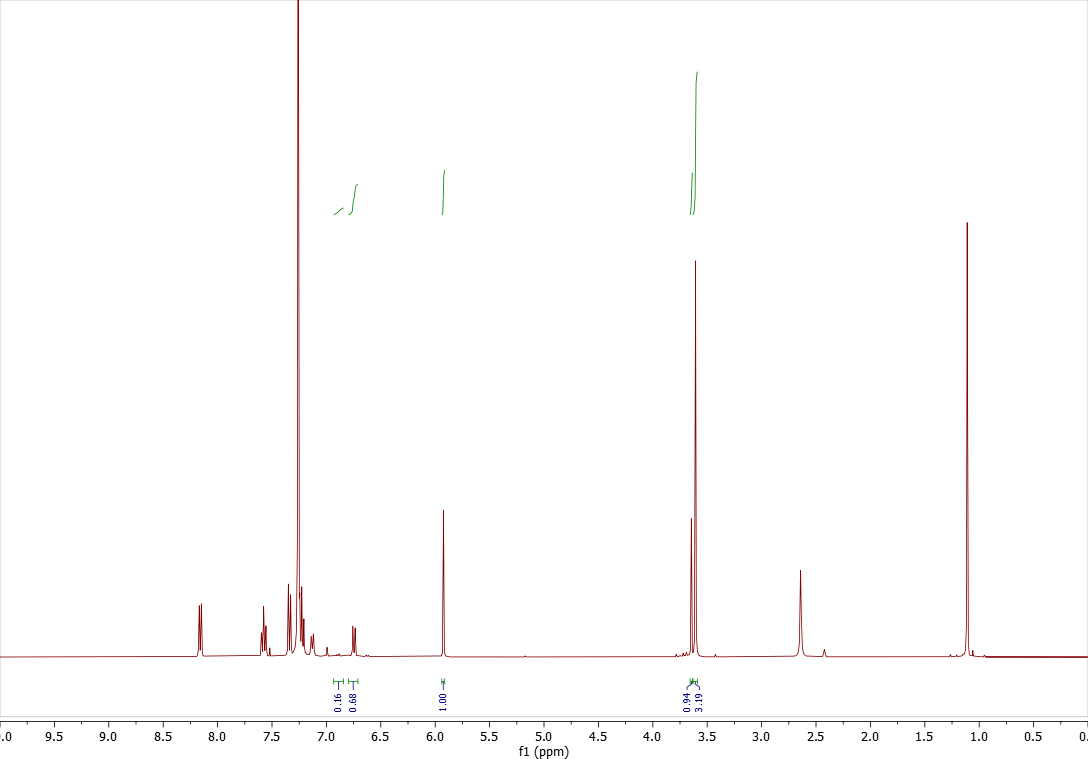


**Figure S4.** Experiment in DMSO-d_6_

**Experiment with TEMPO.** The reaction was set up following the **GP1**, except that TEMPO (1 equiv.) was added. Under those conditions, only trace of the compound **4a** was formed in 34% yield, and no TEMPO adducts were detected. ***It should be mentioned that the current is not very relevant as the TEMPO can be easy reduced by the generated dianion.***

**Figure S5.** Reaction performed in presence of TEMPO

**Experiment with ortho-allyloxybromobenzene.** The reaction was set up following the **GP1**. **Unfortunately, under these conditions, we observed complete degradation of the starting material, with no significant formation of the desired compounds.**

**Figure S6.** Reaction performed in presence of TEMPO

# Computational Details

Geometry optimizations were performed using Gaussian 16 (Revision C01)^13^ at the PBE0 level of hybrid density functional theory,^14^ with inclusion of D3(bj) corrections in the optimization process.^15,16^ The geometries of all located extrema are given as xyz coordinates data in a separate file (Geom.xyz) in the SI. The atoms were represented by an svp basis set.^17^ The solvent (dmso) influence was taken into consideration in the optimization process and through single-point calculations on the gas-phase optimized geometries with SCRF calculations within the SMD model.^18^ For the SCRF single-point calculations, the atoms were treated with a def2-qzvp basis set.^19^ All energies reported are Gibbs free energies obtained at 298 K and 1atm using a procedure described by Ariai and Gellrich in a recent paper to better described the entropy penalty for associative reactions.^20^ The procedure described by equation 14 in Ref 20 has been considered : E_elst_ is obtained in single point calculations with implicit inclusion of the solvent (dmso) by substracting the non-electrostatic component from the converged energy. The entropy contribution of each species is computed from the frequency calculations output using a script obtained from the authors of reference 20.

# References

# Crude NMR Spectra of volatile compounds 4 and 6


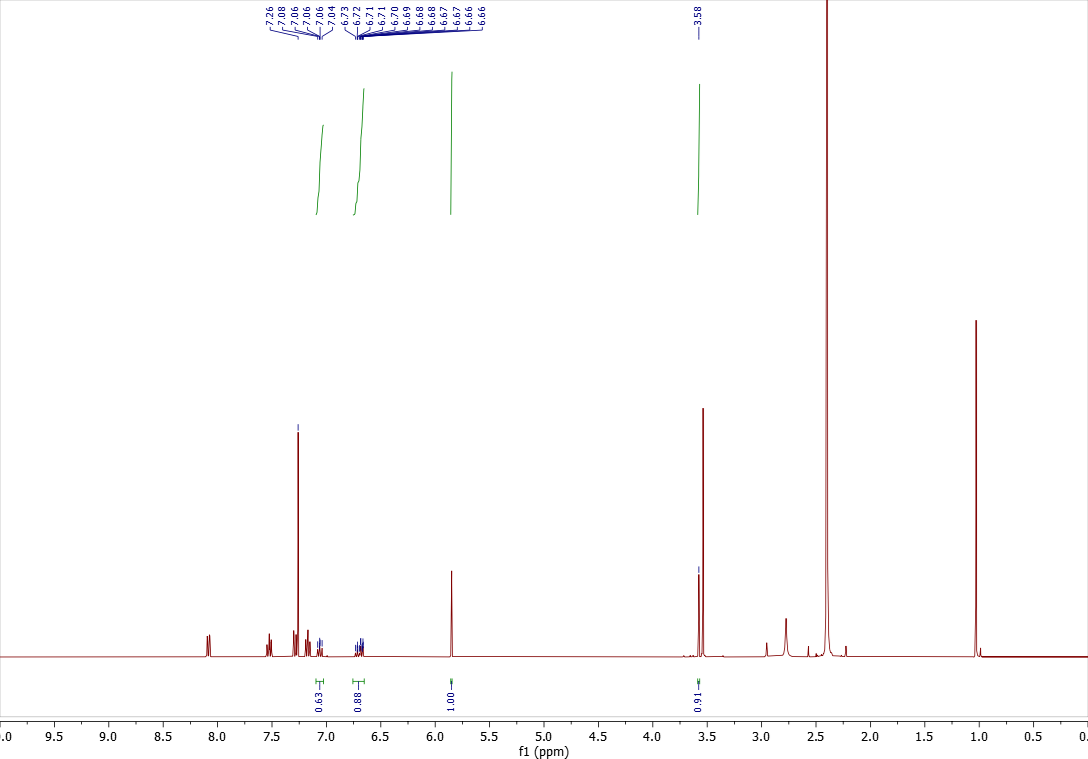


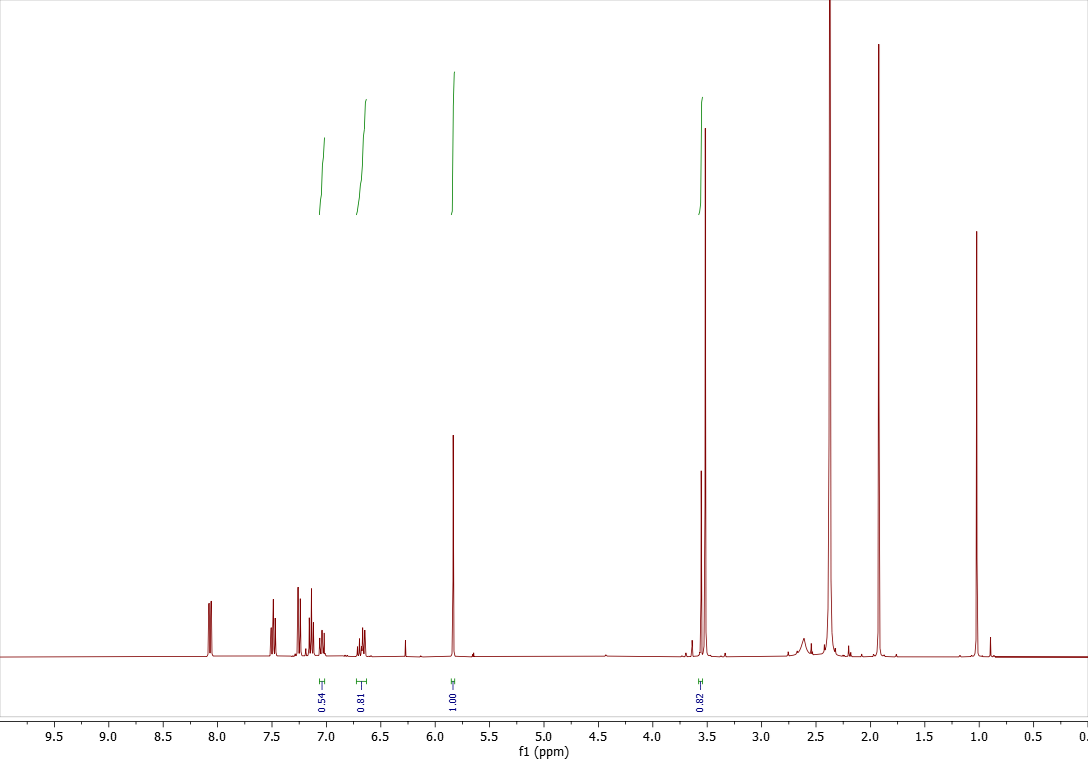


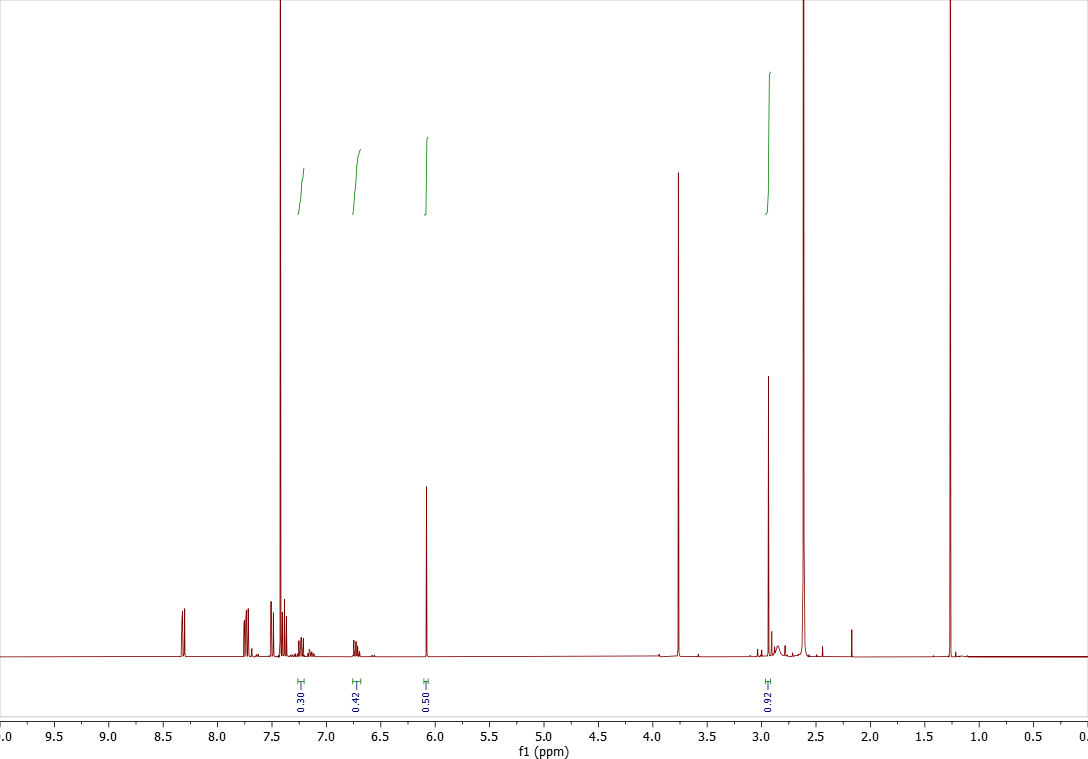


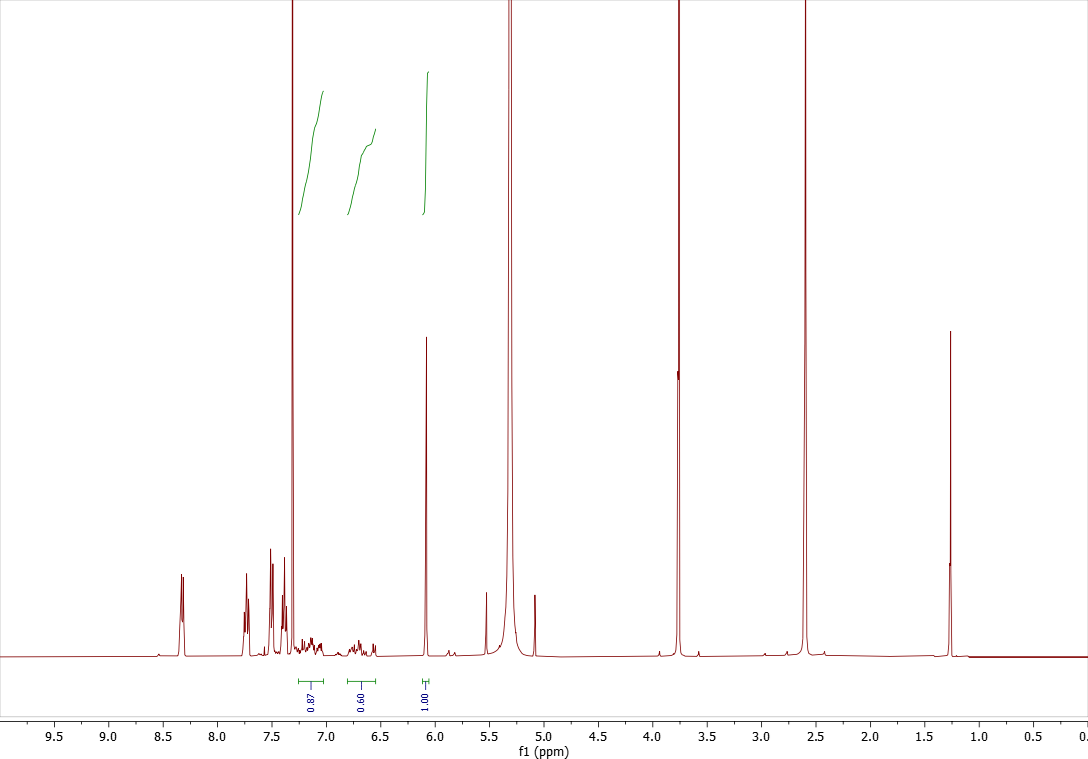


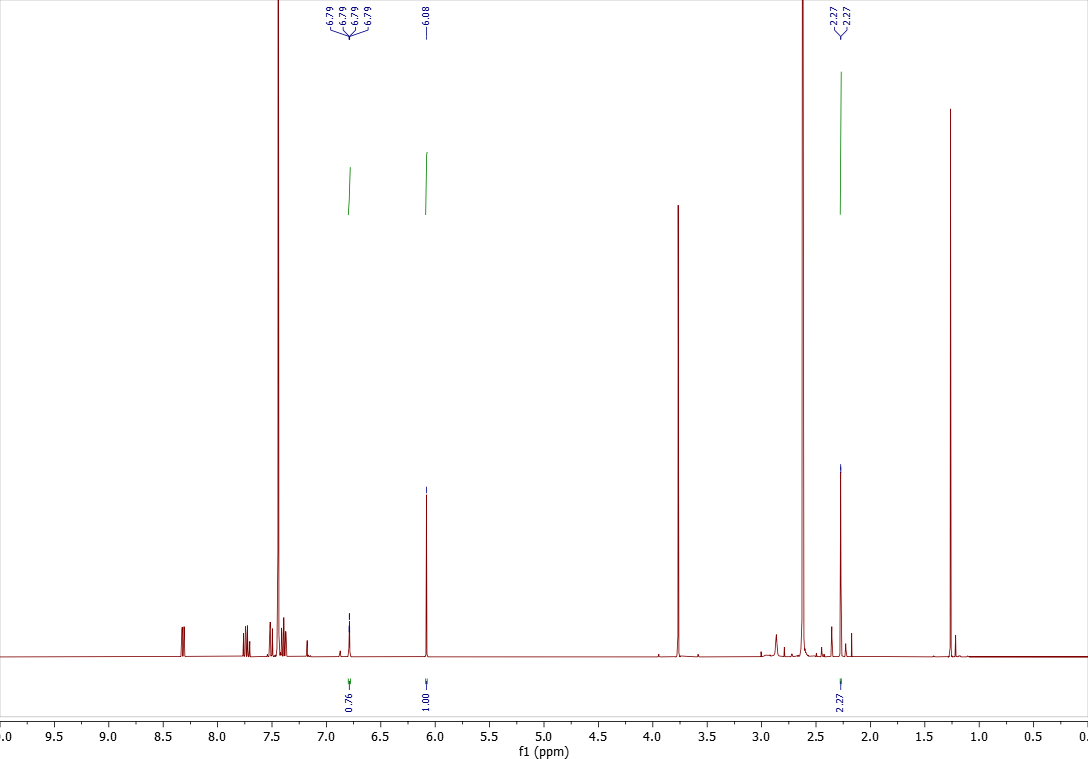


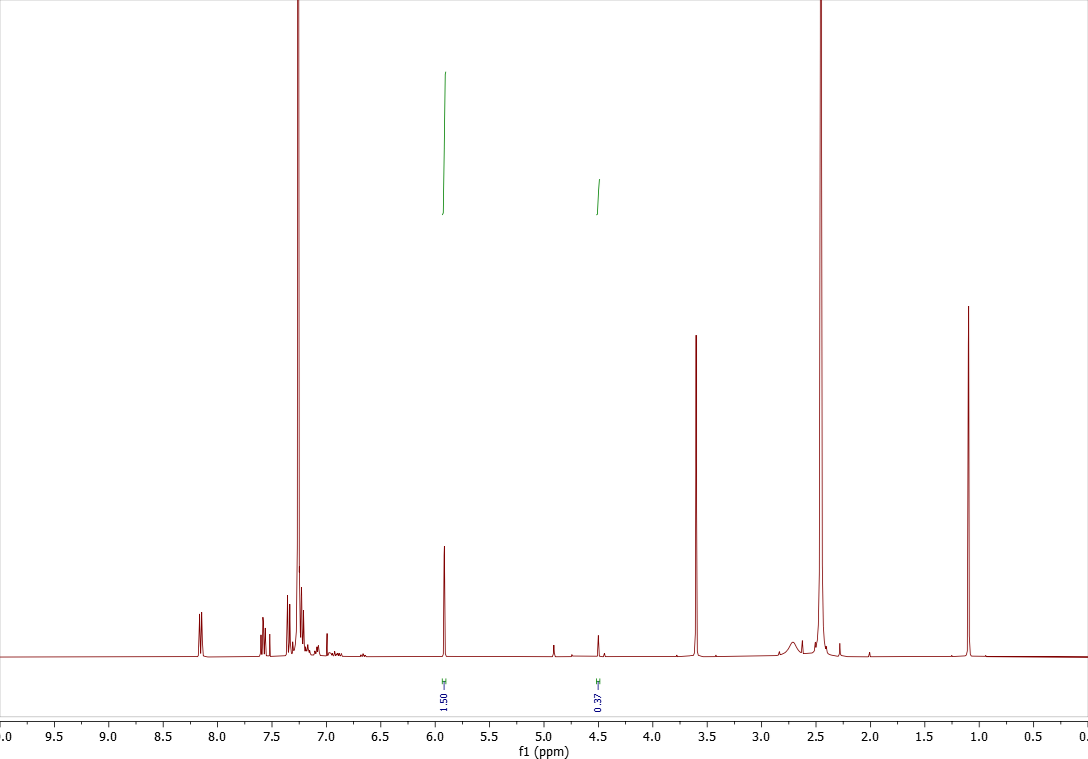


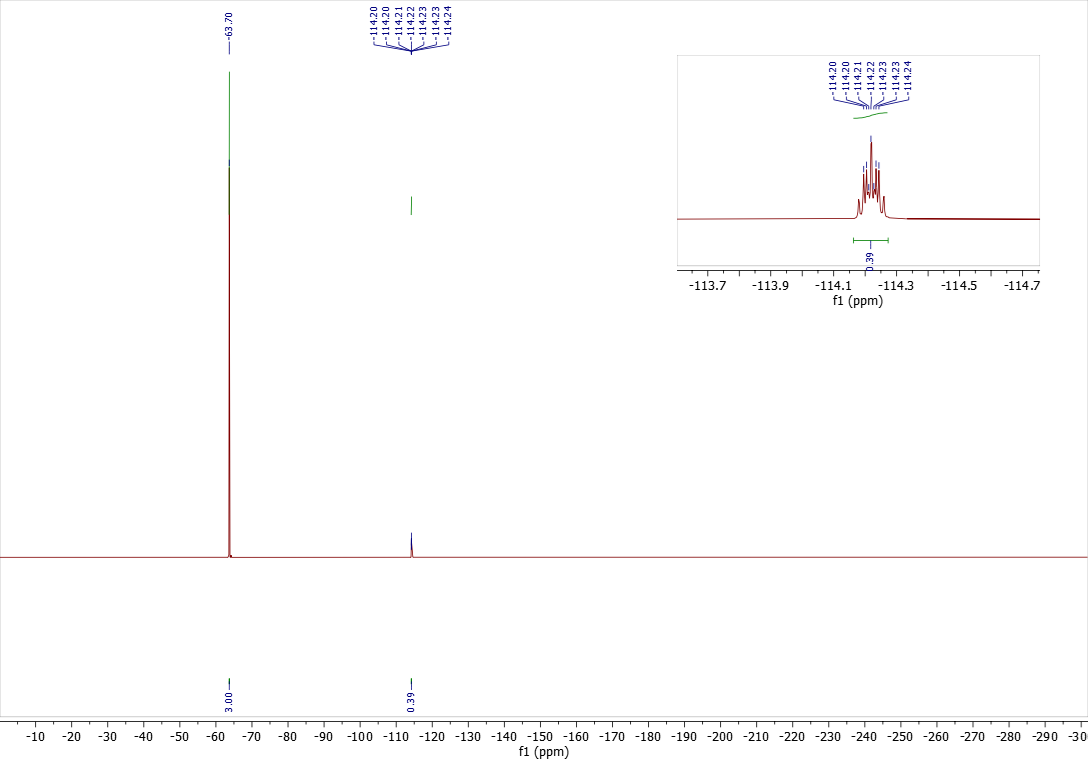


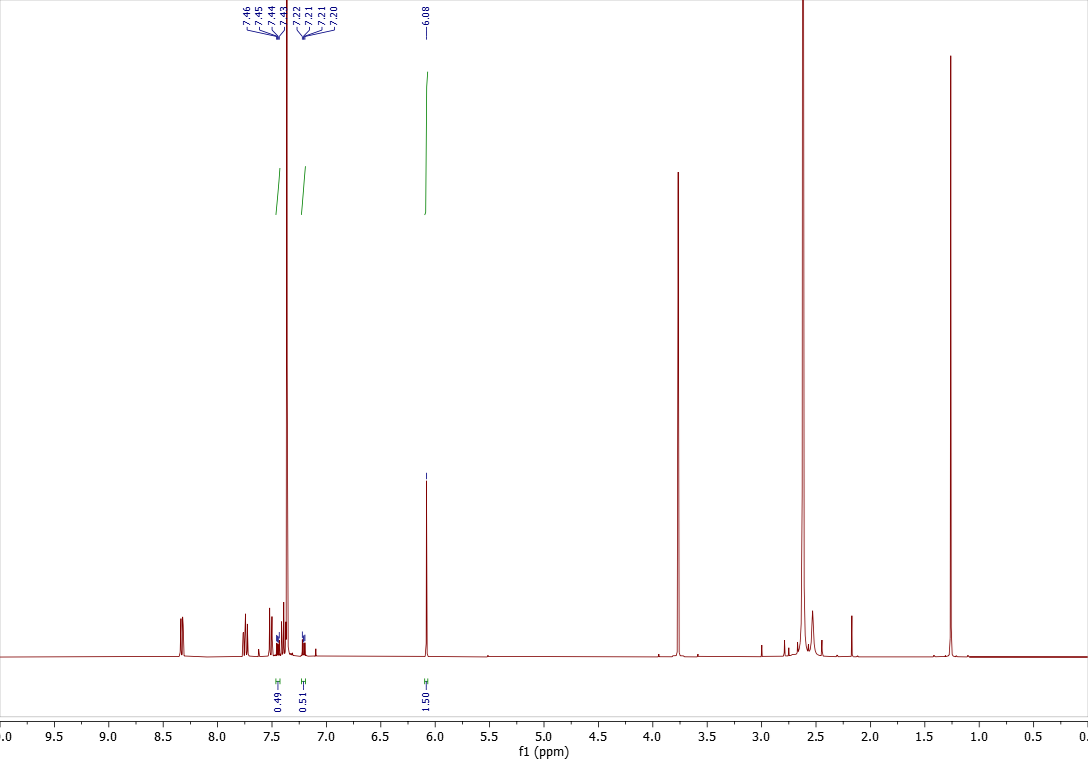


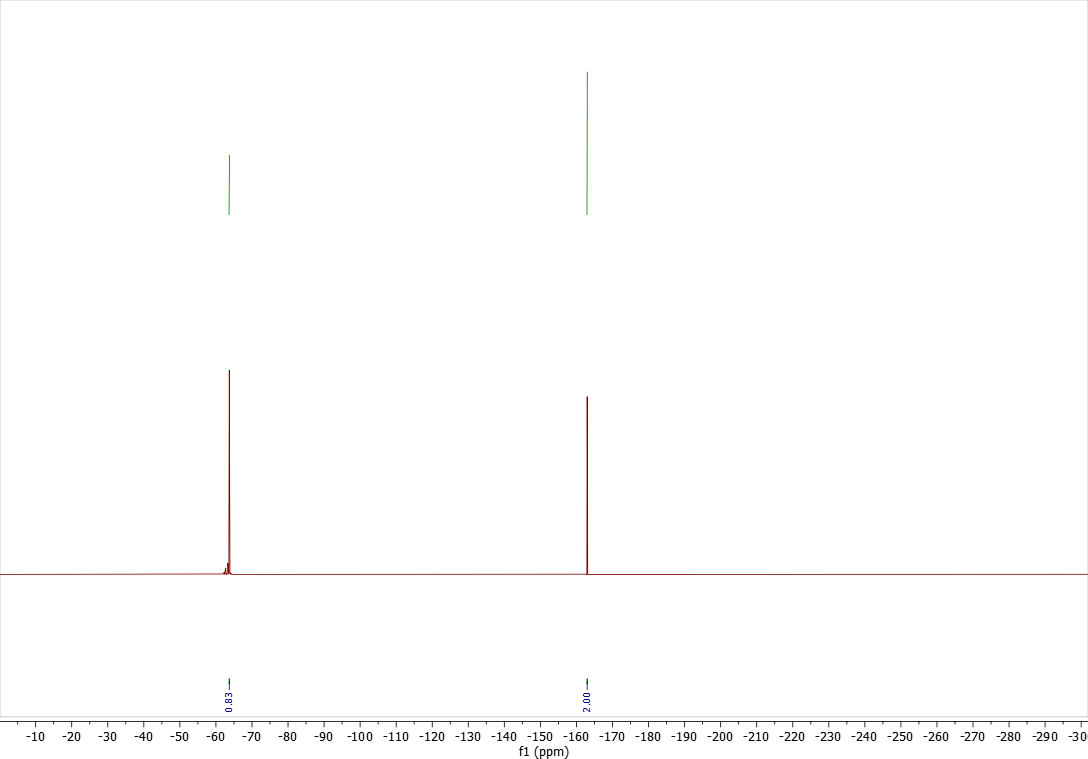


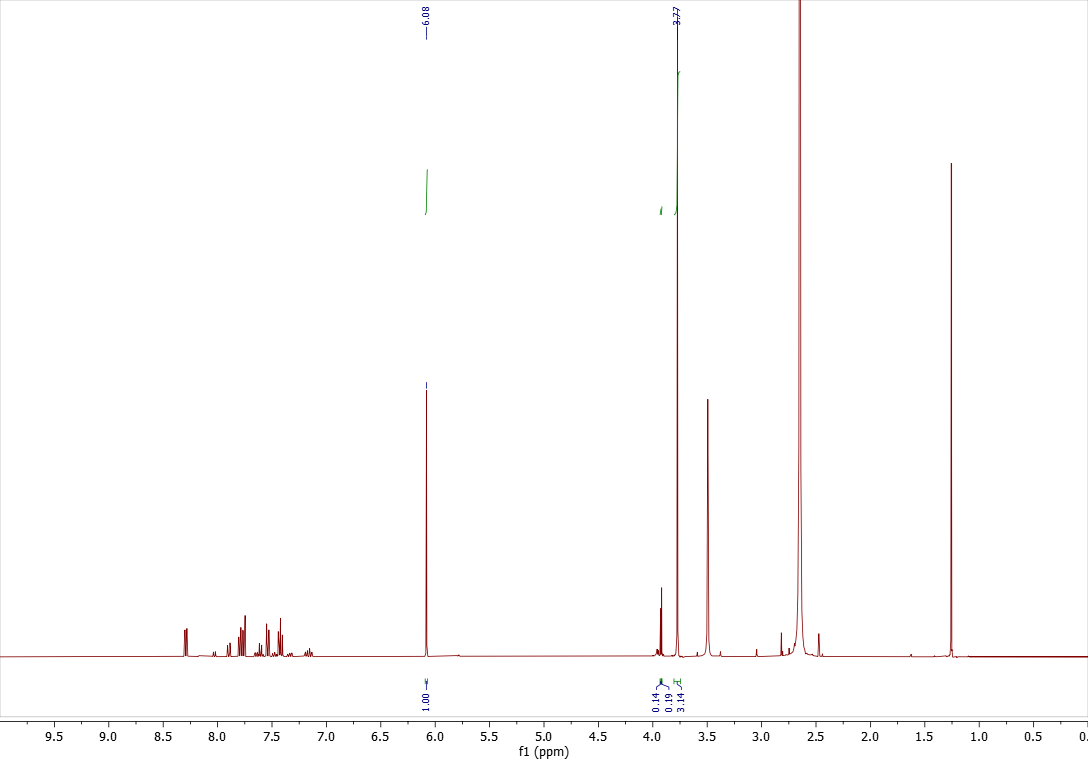


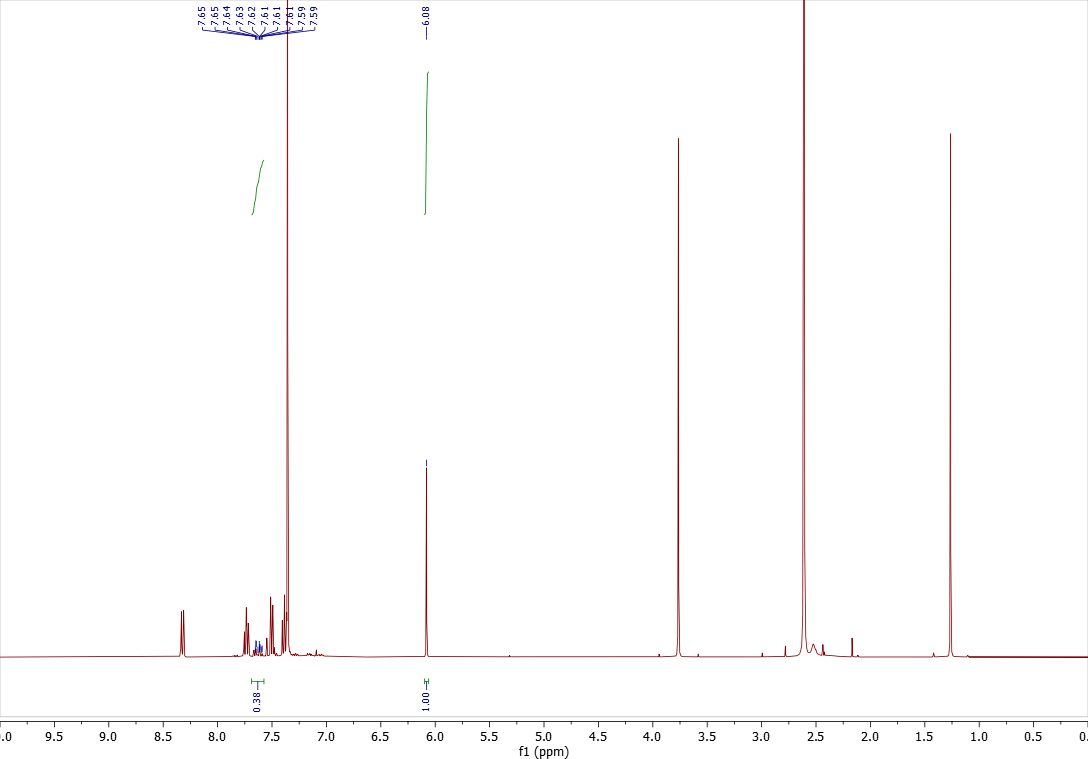


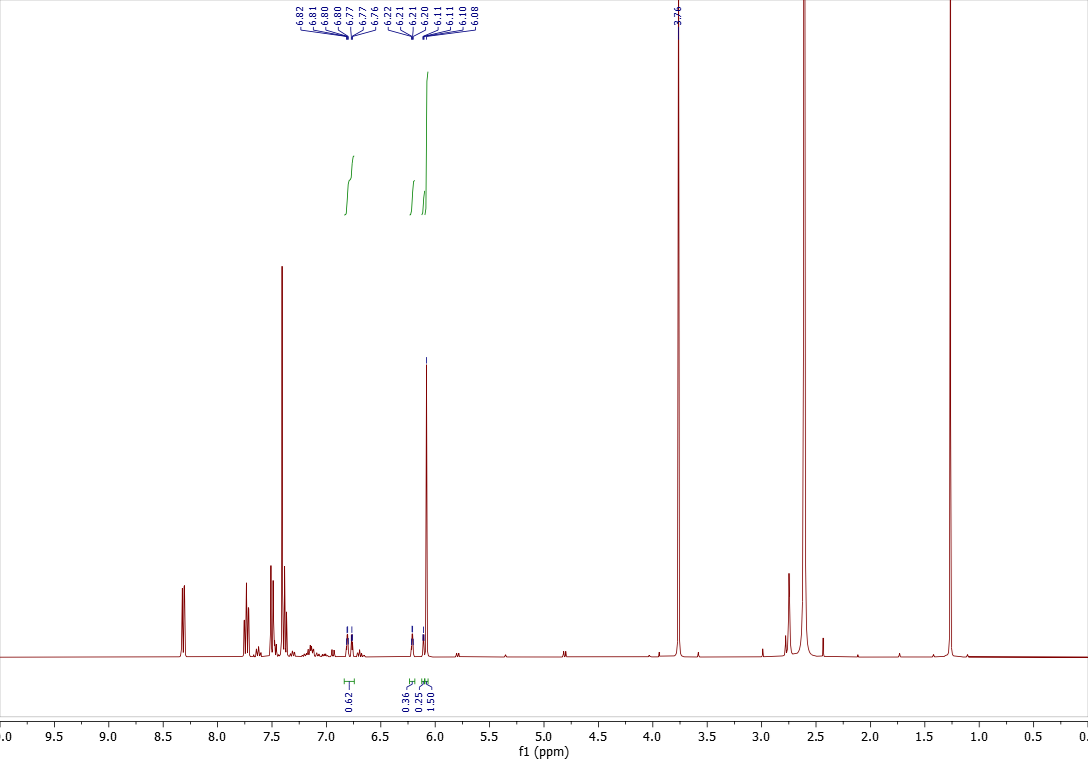


# NMR Spectra of compounds 4 and 6


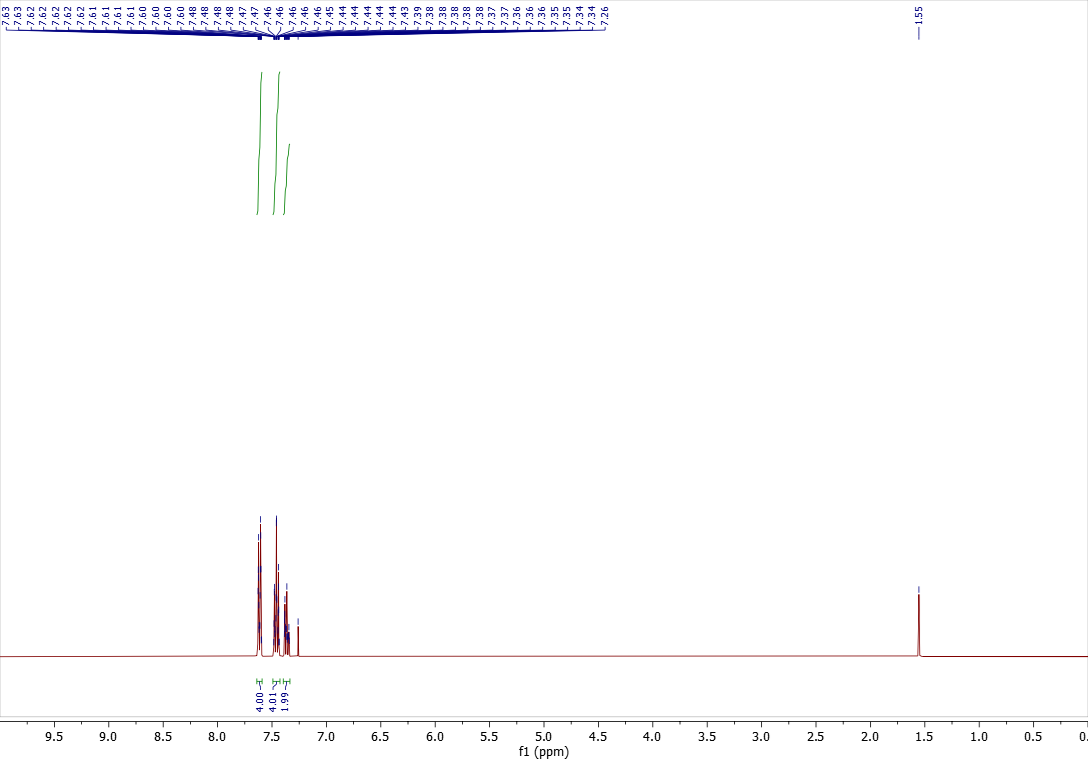


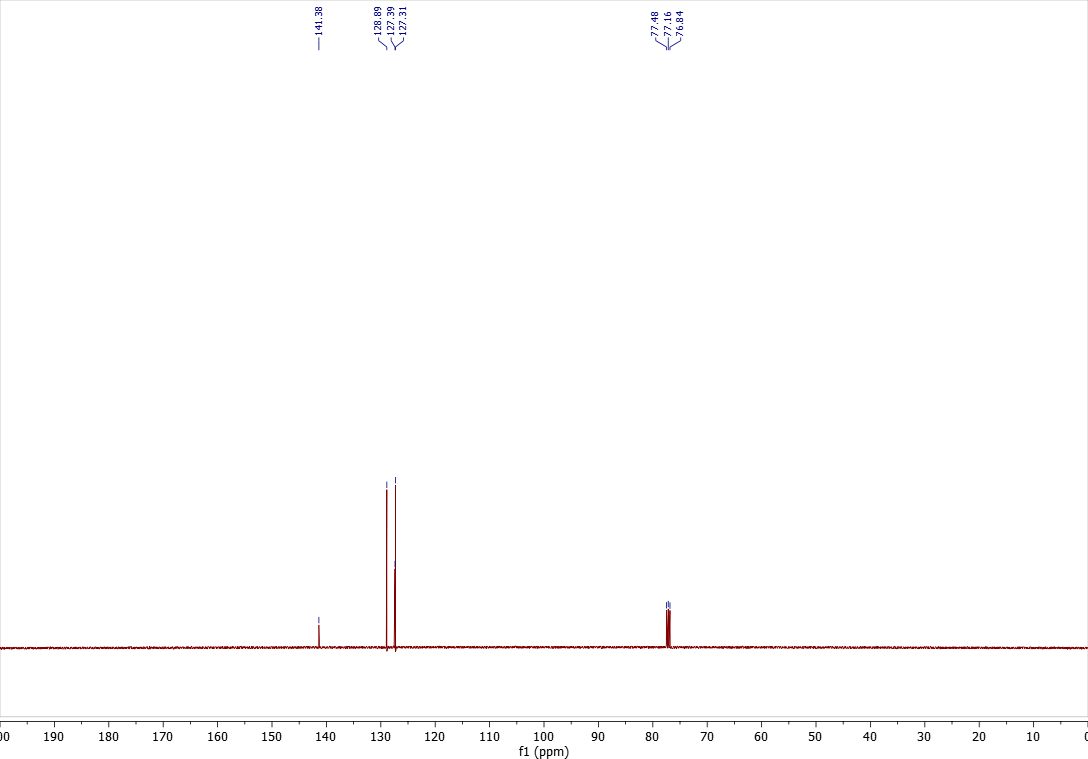


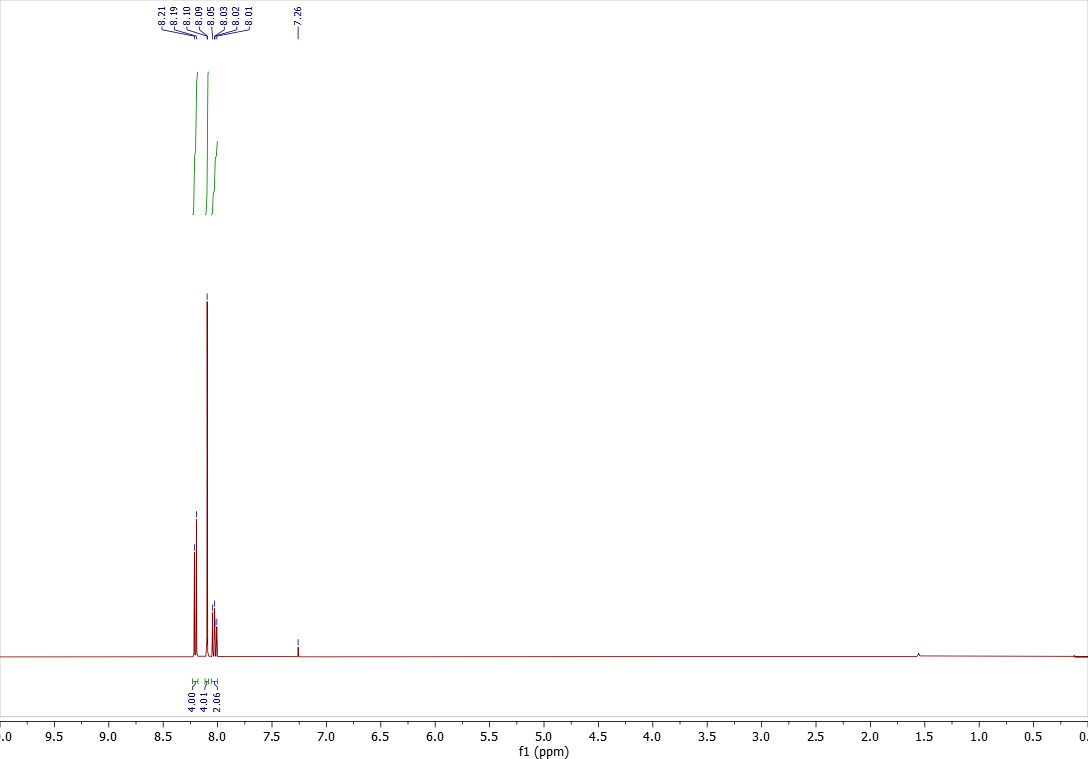


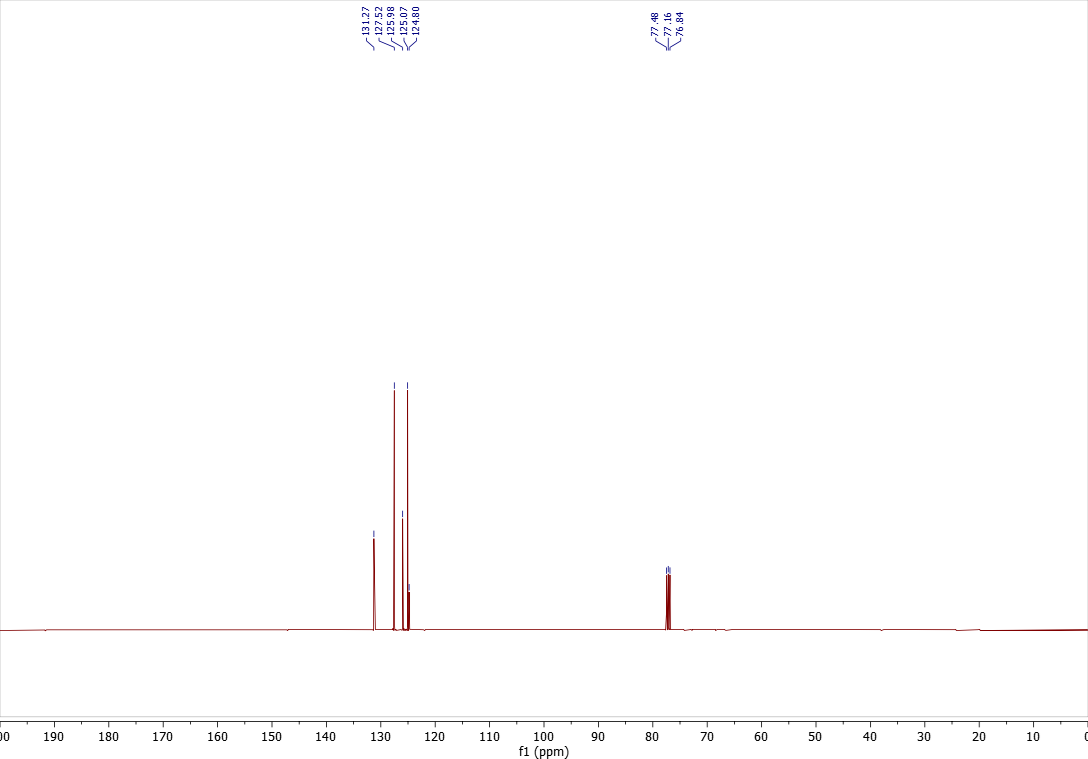


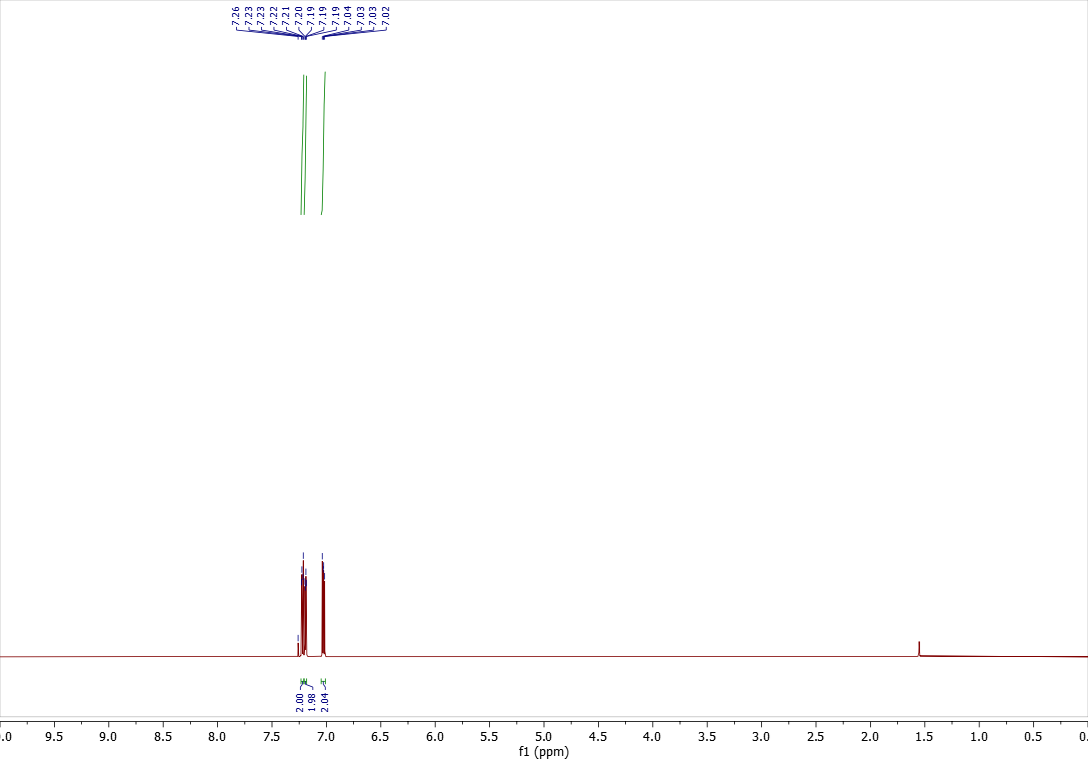


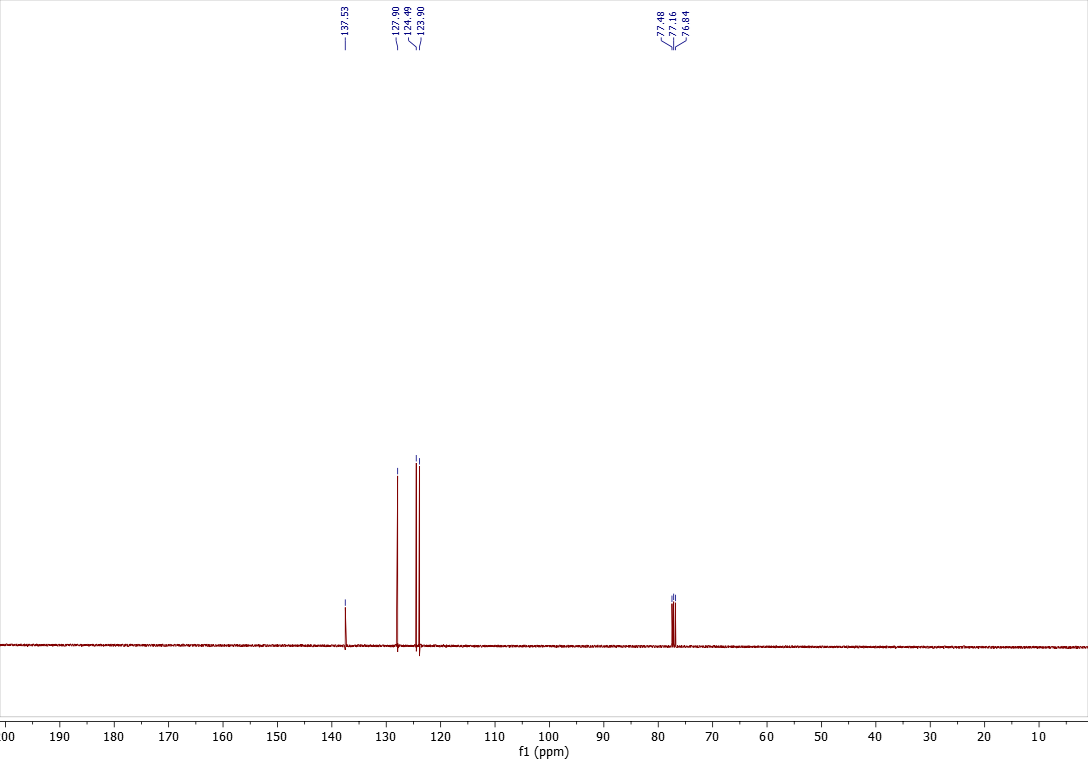


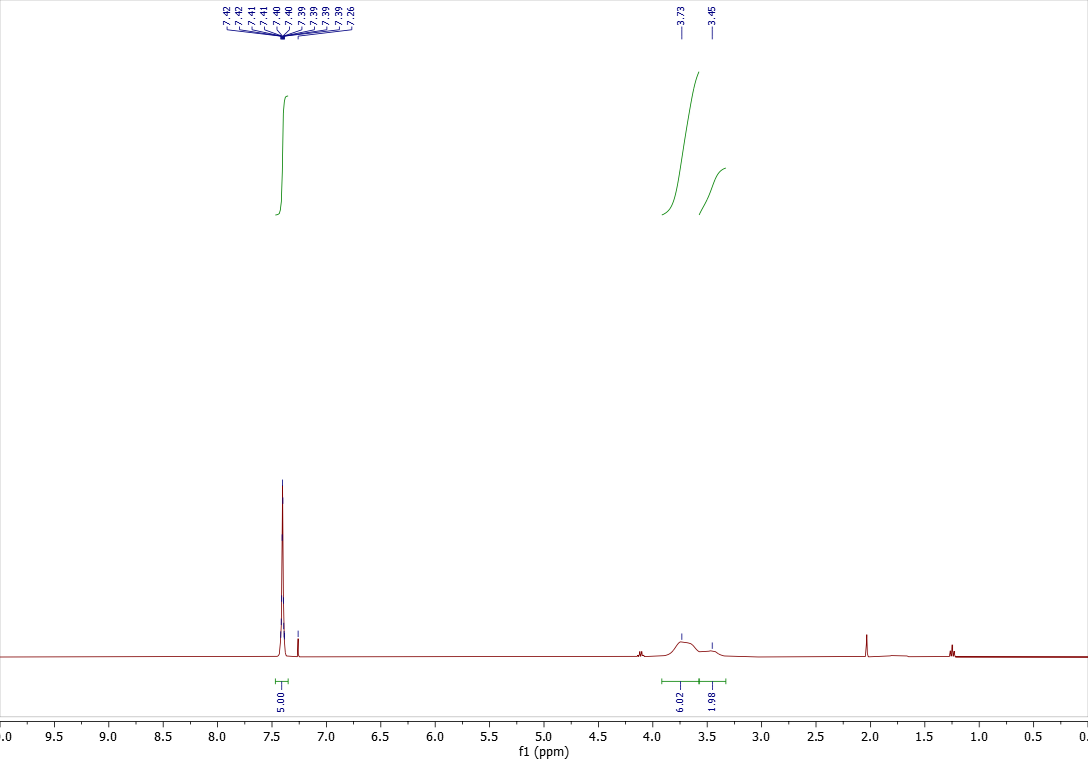


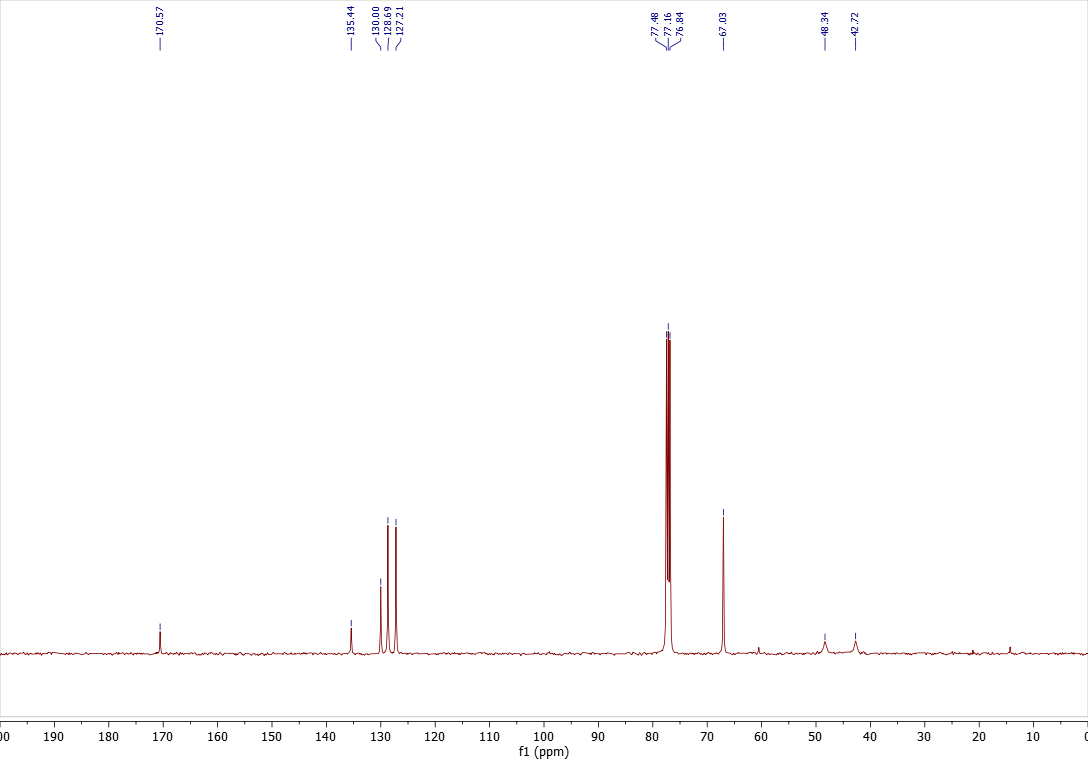


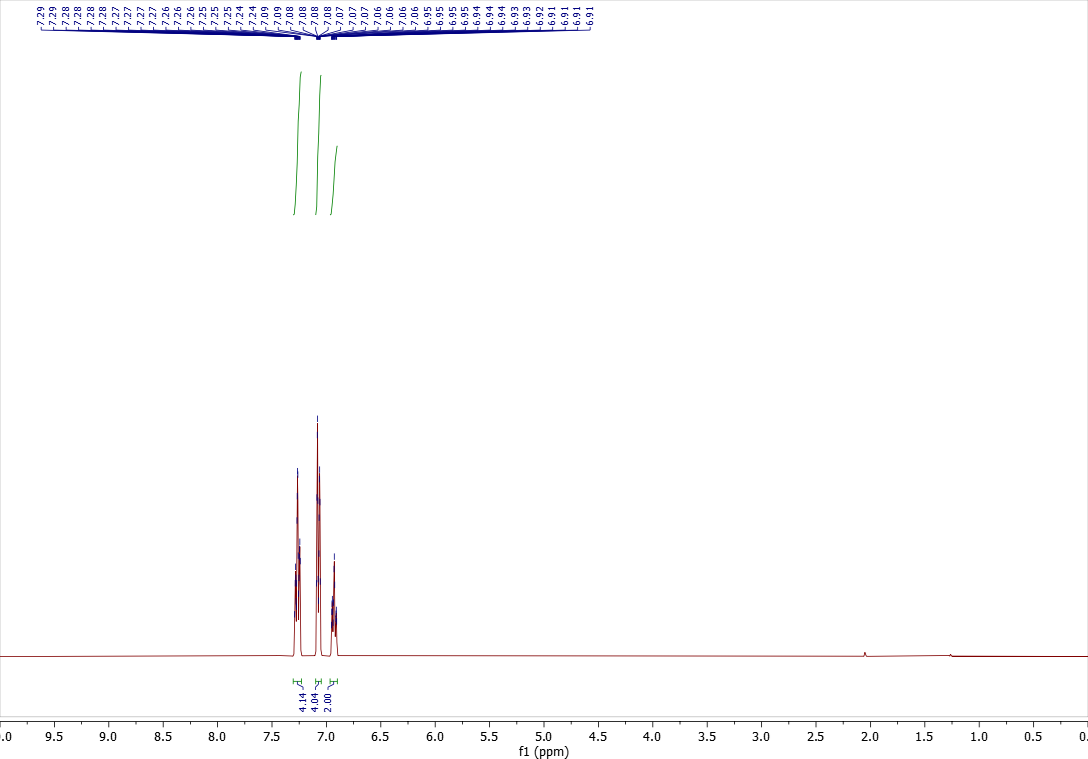


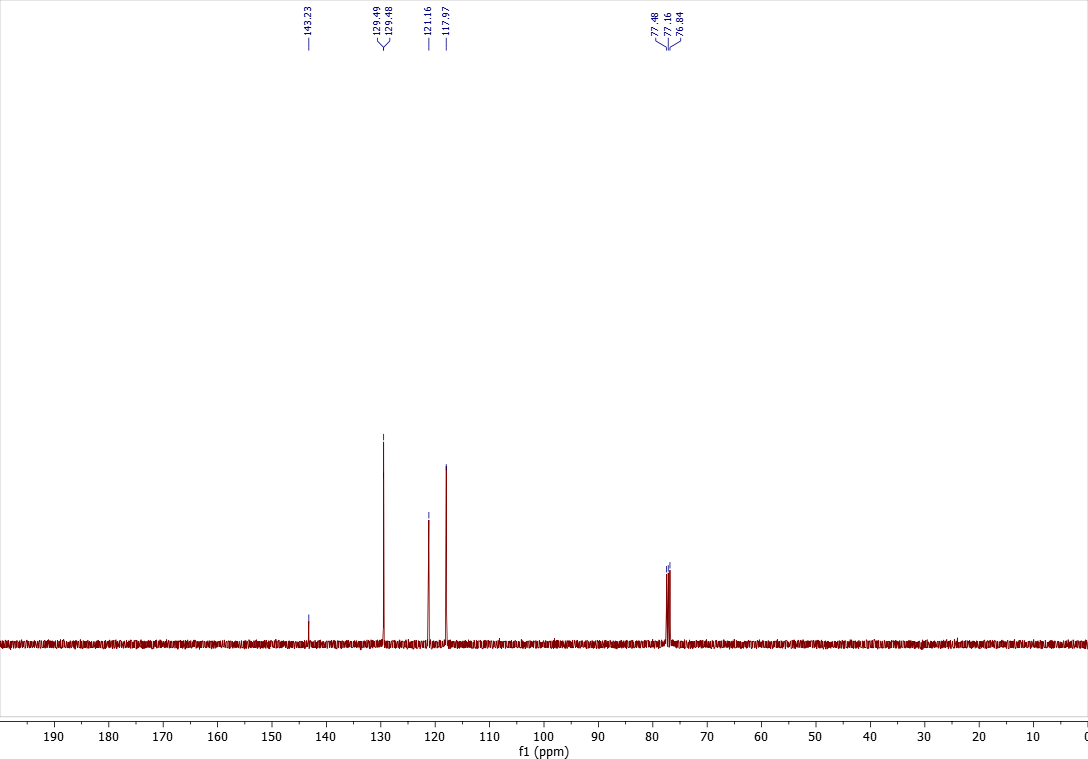


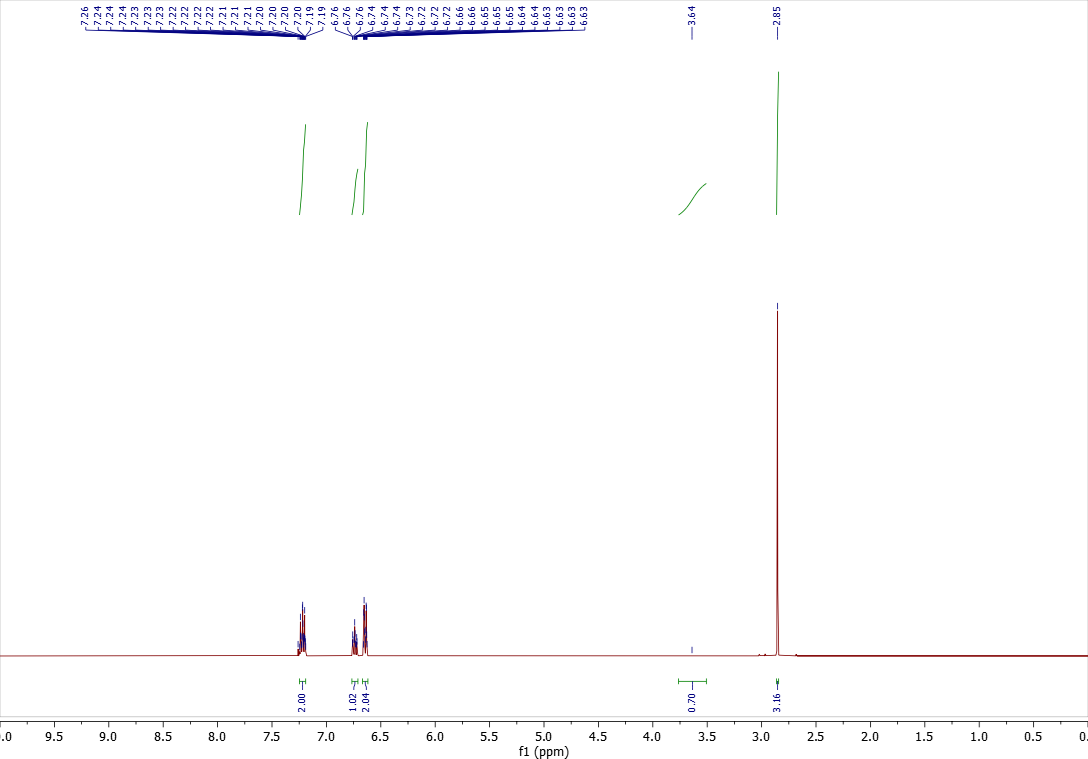


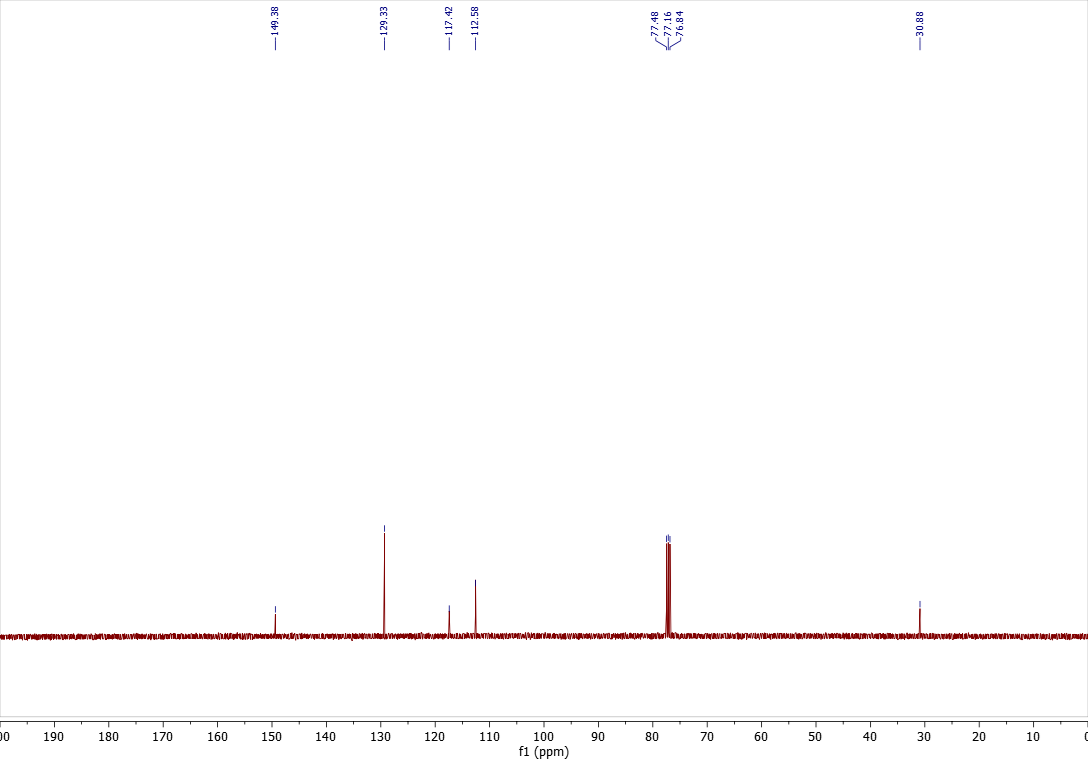


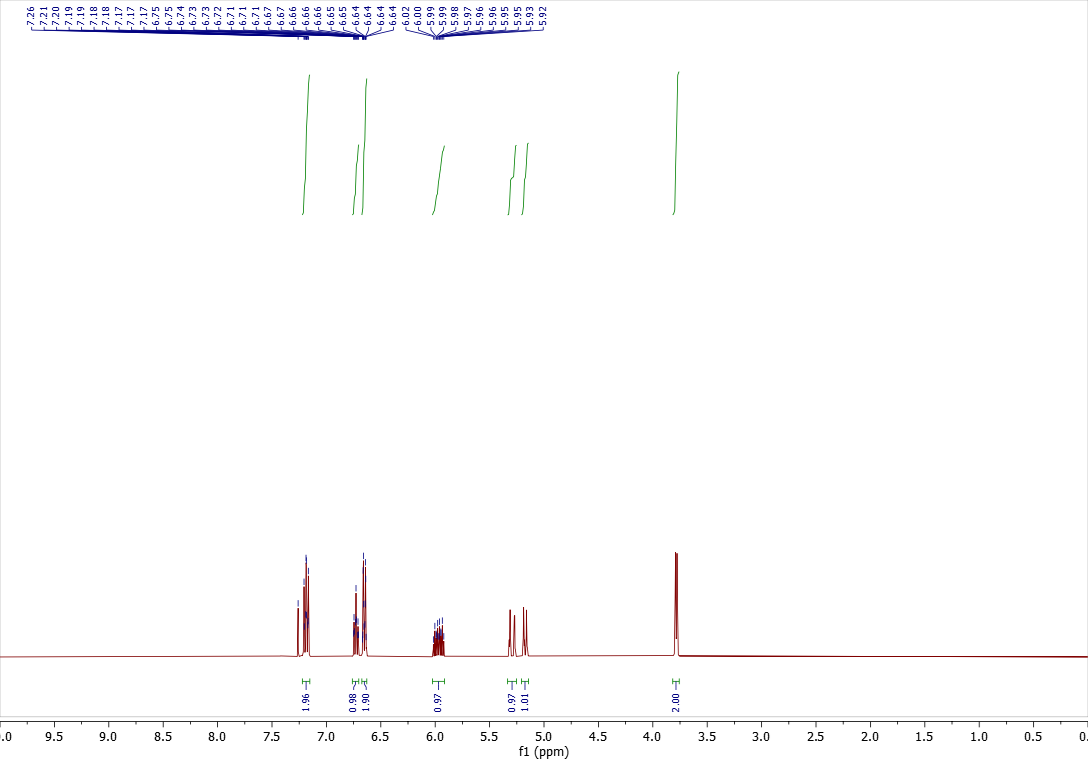


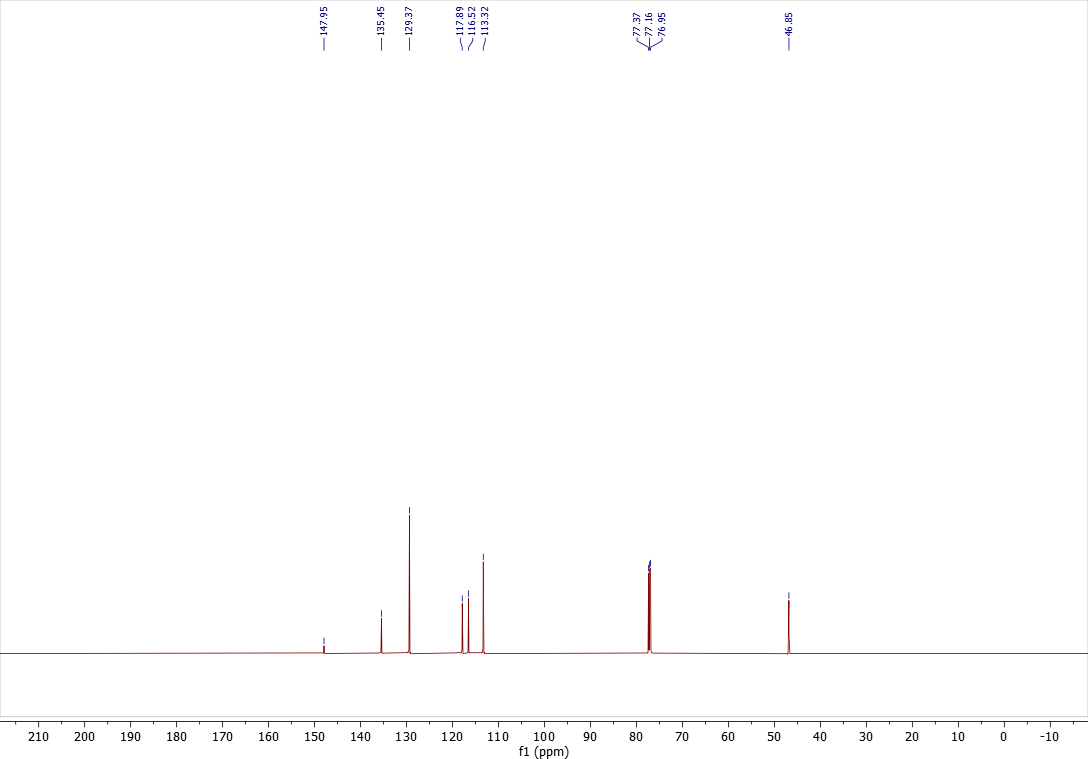


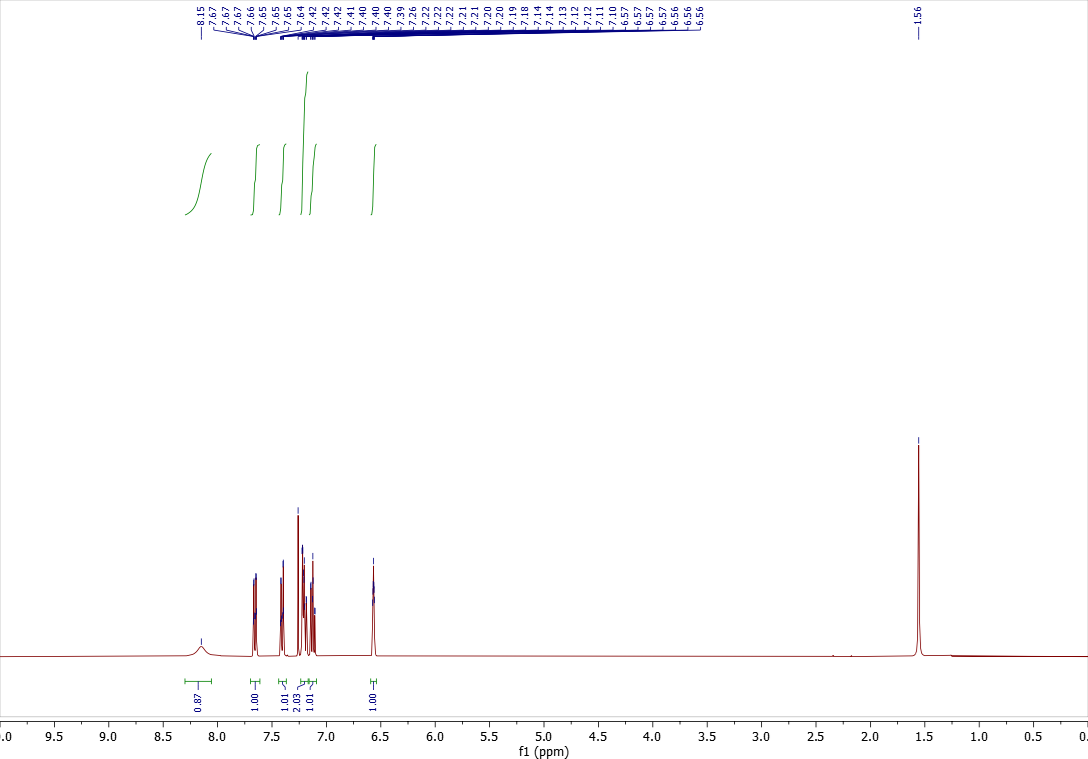


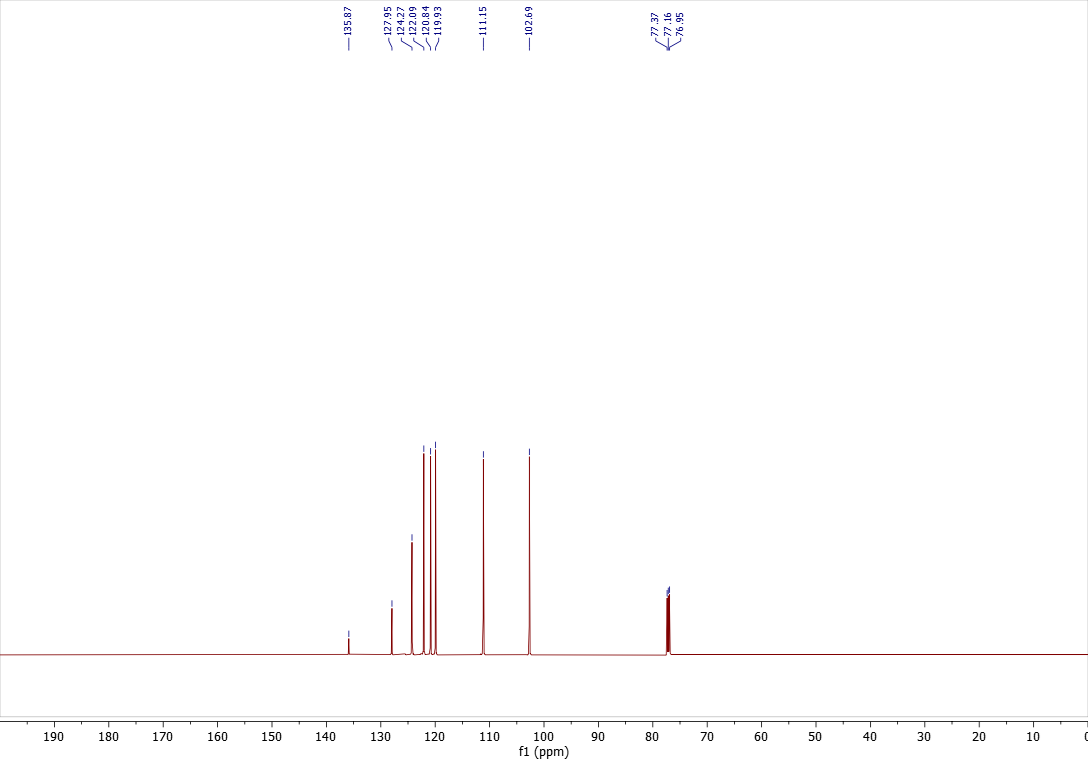


1. D. M. Heard, M. C. Lessard, D. G. Hall, *Angew. Chem. Int. Ed.* **2025**, *64*, e202507571. [↑](#endnote-ref-1)
2. Q. Shen, K. Cao, X. Chen, X. Li, N. Zhang, Y.-B. Miao, J. Li, *Green Chem.* **2023**, *25*, 9665–9671. [↑](#endnote-ref-2)
3. I. A. MacKenzie, L. Wang, N. P. R. Onuska, O. F. Williams, K. Begam, A. M. Moran, B. D. Dunietz, D. A. Nicewicz, *Nature* **2020**, *580*, 76–80. [↑](#endnote-ref-3)
4. T. Igarashi, A. Haito, N. Chatani, M. Tobisu, *ACS Catal.* **2018**, *8*, 7475–7483. [↑](#endnote-ref-4)
5. Akanksha, D. Maiti, *Green Chem.* **2012**, *14*, 2314–2320. [↑](#endnote-ref-5)
6. H. Ji, L.-Y. Wu, J.-H. Cai, G.-R. Li, N.-N. Gan, Z.-H. Wang, *RSC Adv.* **2018**, *8*, 13643–13648. [↑](#endnote-ref-6)
7. T. Ben Halima, J. Masson-Makdissi, S. G. Newman, *Angew. Chem. Int. Ed Engl.* **2018**, *57*, 12925–12929. [↑](#endnote-ref-7)
8. D. S. Raghuvanshi, A. K. Gupta, K. N. Singh, *Org. Lett.* **2012**, *14*, 4326–4329. [↑](#endnote-ref-8)
9. A. Lator, S. Gaillard, A. Poater, J.-L. Renaud, *Org. Lett.* **2018**, *20*, 5985–5990. [↑](#endnote-ref-9)
10. M. Rauser, C. Ascheberg, M. Niggemann, *Angew. Chem. Int. Ed Engl.* **2017**, *56*, 11570–11574. [↑](#endnote-ref-10)
11. J. Wu, D. Talwar, S. Johnston, M. Yan, J. Xiao, *Angew. Chem. Int. Ed Engl.* **2013**, *52*, 6983–6987. [↑](#endnote-ref-11)
12. D. Nicewicz, H. Roth, N. Romero, *Synlett* **2015**, *27*, 714–723.

    ^13^ M. J. Frisch, G. W. Trucks, H. B. Schlegel, G. E. Scuseria, M. A. Robb, J. R. Cheeseman, G. Scalmani, V. Barone, G. A. Petersson, H. Nakatsuji, X. Li, M. Caricato, A. V. Marenich, J. Bloino, B. G. Janesko, R. Gomperts, B. Mennucci, H. P. Hratchian, J. V. Ortiz, A. F. Izmaylov, J. L. Sonnenberg, D. Williams-Young, F. Ding, F. Lipparini, F. Egidi, J. Goings, B. Peng, A. Petrone, T. Henderson, D. Ranasinghe, V. G. Zakrzewski, J. Gao, N. Rega, G. Zheng, W. Liang, M. Hada, M. Ehara, K. Toyota, R. Fukuda, J. Hasegawa, M. Ishida, T. Nakajima, Y. Honda, O. Kitao, H. Nakai, T. Vreven, K. Throssell, J. Montgomery, J. A., J. E. Peralta, F. Ogliaro, M. J. Bearpark, J. J. Heyd, E. N. Brothers, K. N. Kudin, V. N. Staroverov, T. A. Keith, R. Kobayashi, J. Normand, K. Raghavachari, A. P. Rendell, J. C. Burant, S. S. Iyengar, J. Tomasi, M. Cossi, J. M. Millam, M. Klene, C. Adamo, R. Cammi, J. W. Ochterski, R. L. Martin, K. Morokuma, O. Farkas, J. B. Foresman, D. J. Fox, **2016**.

    ^14^ C. Adamo, V. Barone, *J. Chem. Phys.* **1999**, *110*, 6158–6170.

    ^15^ S. Grimme, J. Antony, S. Ehrlich, H. Krieg, J*. Chem. Phys.* **2010**, *132*, 154104.

    ^16^ S. Grimme, S. Ehrlich, L. Goerigk, *J. Comput. Chem.* 2011, *32,* 1456–1465.

    ^17^ A. Schäfer, H. Horn, R. Ahlrichs, *J. Chem. Phys.* **1992**, *97*, 2571–2577.

    ^18^ A. V. Marenich, C. J. Cramer, D. G. Truhlar*, J. Phys. Chem. B*. **2009**, *113*, 6378–6396.

    ^19^ F. Weigend, R. Ahlrichs, *Phys. Chem. Chem. Phys.* **2005**, *7*, 3297–3305.

    ^20^ J. Ariai, U. Gellrich, *Phys. Chem. Chem. Phys.* **2023**, *25*, 14005-14015. [↑](#endnote-ref-12)
